# Crohn's lymphoid aggregates with endothelial clusters colocalise with submucosal fibrosis in fibrostenosing Crohn's disease

**DOI:** 10.1002/path.70019

**Published:** 2026-02-05

**Authors:** Michael Glinka, Gregory J Wickham, Francesca Nadalin, Kathryn J Kirkwood, Helen Caldwell, Mike Wicks, Bill Hill, Derek Houghton, Mehran Sharghi, Amirhosein Kefayat, Bernard Haggarty, Albert Burger, Richard A Baldock, David J Adams, Irene Papatheodorou, Peter Bankhead, Shahida Din, Mark J Arends

**Affiliations:** ^1^ Edinburgh Pathology, CRUK Scotland Centre, Institute of Genetics & Cancer University of Edinburgh Edinburgh UK; ^2^ Earlham Institute Norwich UK; ^3^ EBI, EMBL, Genome Campus, Hinxton Cambridge UK; ^4^ Department of Pathology Western General Hospital, NHS Lothian Edinburgh UK; ^5^ Heriot‐Watt University Edinburgh UK; ^6^ Edinburgh IBD Unit, Western General Hospital, NHS Lothian Edinburgh UK; ^7^ Wellcome Sanger Institute Genome Campus, Hinxton Cambridge UK; ^8^ Medical School University of East Anglia Norwich UK; ^9^ Centre for Genomic & Experimental Medicine, Institute of Genetics & Cancer University of Edinburgh Edinburgh UK

**Keywords:** Crohn's disease, Fibrostenotic lesion, QuPath, Crohn's lymphoid aggregates

## Abstract

Crohn's disease (CD) involves chronic transmural inflammation of the intestines, leading to progressive wall fibrosis with stenosis and luminal obstruction, predominantly in the terminal ileum. Fibrosis is a significant therapeutic challenge, thus improved understanding of localisation, cellular composition, and cell–cell interactions in CD fibrostenosing lesions (FSLs) may identify potential targetable pathways. Using CD FSL patient resection samples, we identify and quantify novel pathological changes in structure, collagen, and cell numbers for each ileal layer (mucosa, muscularis mucosae, submucosa, muscularis propria, serosa). In addition, fresh resection ileal samples were single‐cell RNA (scRNA)‐sequenced, validating the cell types and cell–cell interactions. We found significantly increased collagenous fibrosis expansion, significantly increased infiltration of lymphocytes, macrophages, endothelium, and Crohn's lymphoid aggregates (CLAs) in all layers, except for the ulcerated mucosa. Importantly, endothelial cells accumulate in clusters around CLAs, and scRNA‐seq data demonstrated ligand–receptor intercellular signalling interactions between endothelium, B and T lymphocytes, macrophages, and myofibroblasts via multiple pathways that included GAS, SELL, and SELPLG, among many others. The highest levels of fibrotic collagen and CLAs with accumulated endothelium were observed in submucosa, followed by serosa, demonstrating colocalisation and correlation of endothelial‐CLAs with collagen that is consistent with CLAs having a role in promoting collagenous fibrosis that requires further investigation. © 2026 The Author(s). *The Journal of Pathology* published by John Wiley & Sons Ltd on behalf of The Pathological Society of Great Britain and Ireland.

## Introduction

Crohn's disease (CD) is characterised by inflammation along the entire gastrointestinal (GI) tract and is becoming more prevalent globally. CD or ‘diseases’, as has been postulated [1], are associated with multiple genetic factors [2] identified as single nucleotide polymorphisms (SNPs), which influence CD risk but only account for < 26% of the variance and are unable to predict therapeutic response or provide prognostic clinical information [3].

CD is associated with numerous environmental (smoking, stress, others) and intestinal microbiota factors, presenting a complex challenge in deciphering the pathogenic mechanisms of the disease. As the intestinal chronic inflammation progresses and increases in severity, up to half of patients may have fibrosis with a proportion developing a fibrostenotic lesion (FSL), which can cause partial intestinal obstruction [2, 4]. Currently, no treatment is available for fibrosis except for surgical resection or mechanical disruption of the affected segment and surrounding tissue [5], although anti‐fibrotic drugs are under investigation [4]. The majority of CD FSL cases have significant chronic inflammation [2, 5, 6]. The most commonly affected GI tract site for CD FSL is the ileum, accounting for > 75% cases [7].

Of the four ileal layers—mucosa, submucosa, muscularis propria and serosa [8]—in CD, frequent mucosal ulceration compromises the intestinal barrier, enabling luminal antigens to generate an immune response and inflammation. The ulceration with cytokines released from the immune cells leads to increased vascular granulation tissue forming in the mucosa and submucosa propagating the chronic inflammatory response deeper into the other layers [5, 9]. In addition, the released cytokines can trigger epithelial‐to‐mesenchymal transition, which increases the number of fibroblasts and myofibroblasts [10, 11, 12], leading to collagen deposition within the tissue, sometimes forming a fibrotic scar [6, 13, 14]. Analysis of collagen present in FSL showed that it was mostly composed of collagen types I (68%), III (20%), and V (5%) [15, 16]. Additionally, muscularis propria and serosa layers become expanded, with increased adipose tissue in the serosa described as ‘fat wrapping’ or ‘creeping fat’ around the ileal surface at the site of FSLs [2, 17]. FSLs show muscle hyperplasia/hypertrophy as well as fibrosis [18], with overlap between inflammatory and fibrotic processes seen on cross‐sectional imaging of CD fibrosis, as shown in previous studies, but were poorly correlated with the histopathological changes of fibromuscular stenosis [19, 20, 21]. Suboptimal histopathological and radiological characterisation affects prediction of the effects of medical or surgical interventions, which remains a substantial therapeutic challenge [2, 22, 23]. Furthermore, the lack of a validated histopathological index to determine the impact of treatment was addressed in a modified RAND/University of California Los Angeles methodology to standardise histopathological scoring for small bowel strictures in CD [23]. The working group considered three parameters on gross pathology assessment, appropriate and necessary to diagnose a small bowel stricture: an increased small bowel wall thickness, decreased internal circumference, and decreased luminal diameter. Only fibrosis of the submucosa was considered appropriate for the histopathological features necessary to diagnose small bowel strictures [23].

Precise intestinal wall layer annotation is critical to define the spatial aspects of cellular and fibrotic disease heterogeneity. Biopsy studies below the mucosa are often presented as aggregated tissue without distinction by layer [24] or quantification of the changes only within the mucosa and inner submucosa and not throughout all of the wall layers [25, 26, 27, 28, 29, 30, 31, 32, 33, 34].

Here, we present accurate layer‐specific characterisation and quantification (by QuPath [35]) of collagen and immune cells in cases of surgically resected full wall thickness tissue block sections of CD FSL compared with normal control ileum.

## Materials and methods

For additional detailed descriptions of some methods, see the Supplementary Materials and Methods.

### Ethics approval

All tissue was acquired via the National Health Service (NHS) Bioresource under ethics approval 20/ES/0061 (SR1492 and SR1803) (NHS Lothian, UK).

### Tissue acquisition, processing, and imaging

#### Archival tissue acquisition

The 30 archival normal control (NC) ileum (normal ileal tissue from unrelated non‐IBD conditions with no ileal abnormalities) and 30 CD fibrostenosing lesion (FSL) samples from ilea were acquired from NHS Bioresource under ethics approval 20/ES/0061 (SR1492). The tissue was processed and stained with haematoxylin and eosin (H&E), Picro‐Sirius Red (PSR), and immunohistochemical (IHC) stains [using antibodies against smooth muscle actin (SMA), CD3, CD4, CD8, CD20, CD31, and CD68] as per standard protocol (see Supplementary materials and methods). Stained images were scanned using the Hamamatsu Nanozoomer XR Slidescanner (Welwyn Garden City, Hertfordshire, UK) and analysed quantitatively with QuPath [35].

#### Fresh tissue acquisition and dissociation for single‐cell RNA sequencing (scRNA‐seq)

The fresh control (*n* = 4, tissue) and CD FSL (*n* = 3) ileum samples were collected from consenting patients [NHS Bioresource ethics approval 20/ES/0061 (SR1803)]. The tissue was processed and dissociated fresh to acquire single‐cell suspension based on a previously published protocol [36] with modifications (see Supplementary materials and methods).

#### Annotation of intestinal wall layers and quantification of components

Each ileal wall layer (mucosa, muscularis mucosae, submucosa, muscularis propria, and serosa) was manually annotated, and the mucosa annotations were corrected using a pretrained QuPath pixel classifier to ensure correct identification of the mucosal tissue layer without luminal space.

To quantify the amount of collagen present in the different layers of intestinal tissue stained with PSR, a QuPath pixel classifier was trained to detect ‘collagen’ (Collagen, pink), ‘whitespace’ (lipid droplets in fat cells, vascular lumen, and white background) (Negative, blue) and ‘other’ (infiltrating inflammatory or immune cells, red‐blood cells, and muscle tissue) (Other, yellow).

The immunohistochemically identified DAB‐positive cells stained in brown were quantified using the built‐in ‘Positive cell detection’ function of QuPath. For Crohn's lymphoid aggregate (CLA) and granuloma detection, the ‘Density Map’ function in QuPath was used.

### Image data analysis

The data from QuPath were collated using a Python 3 script into .csv files. The results were analysed using R (R‐4.3.3) in RStudio (RStudio 2023.12.1 Build 402). All code for the analysis is available on GitHub: https://github.com/Comparative‐Pathology/GCA_QuPathLayerAnnotations

### scRNA‐seq data analysis

scRNA‐seq data were analysed with CellRanger (9.0.1) using the human reference genome GRCh38. The resulting count matrices were filtered to retain only high‐quality cells (control/normal: 25,472, CD: 32,083 cells; see Supplementary materials and methods). Cell type annotation was performed using a semi‐supervised framework with scvi‐tools (1.3.3). A latent representation was learned on reference annotations from published Gut Cell Atlas datasets [37] with scVI and used to initialise a scANVI model for transfer of reference labels to query data. Joint Uniform Manifold Approximation and Projection (UMAP) embeddings were used to visualise data, and predicted labels were manually refined into curated cell type identities based on canonical marker gene expression. Cell type compositions between conditions were modelled with scCODA (0.1.9) [38], and intercellular communication networks were inferred using CellChat (2.1.1) [39], based on expression patterns of known ligand–receptor pairs.

### Statistical analyses

All data were analysed with the Shapiro–Wilk test, in addition Q–Q plots and density plots were generated to determine the data normality. For non‐normally distributed data, the Wilcoxon rank‐sum and signed‐rank tests were used to determine significant differences between the data from normal/control versus CD FSL for each ileal wall layer. For multiple comparison analysis, the Kruskal–Wallis test with Dunn's test of multiple comparisons were used with Bonferroni *post hoc* adjustment.

## Results

### Quantitative changes in ileal layers, smooth muscle components, and collagen deposition in CD FSL

**Figure 1:**
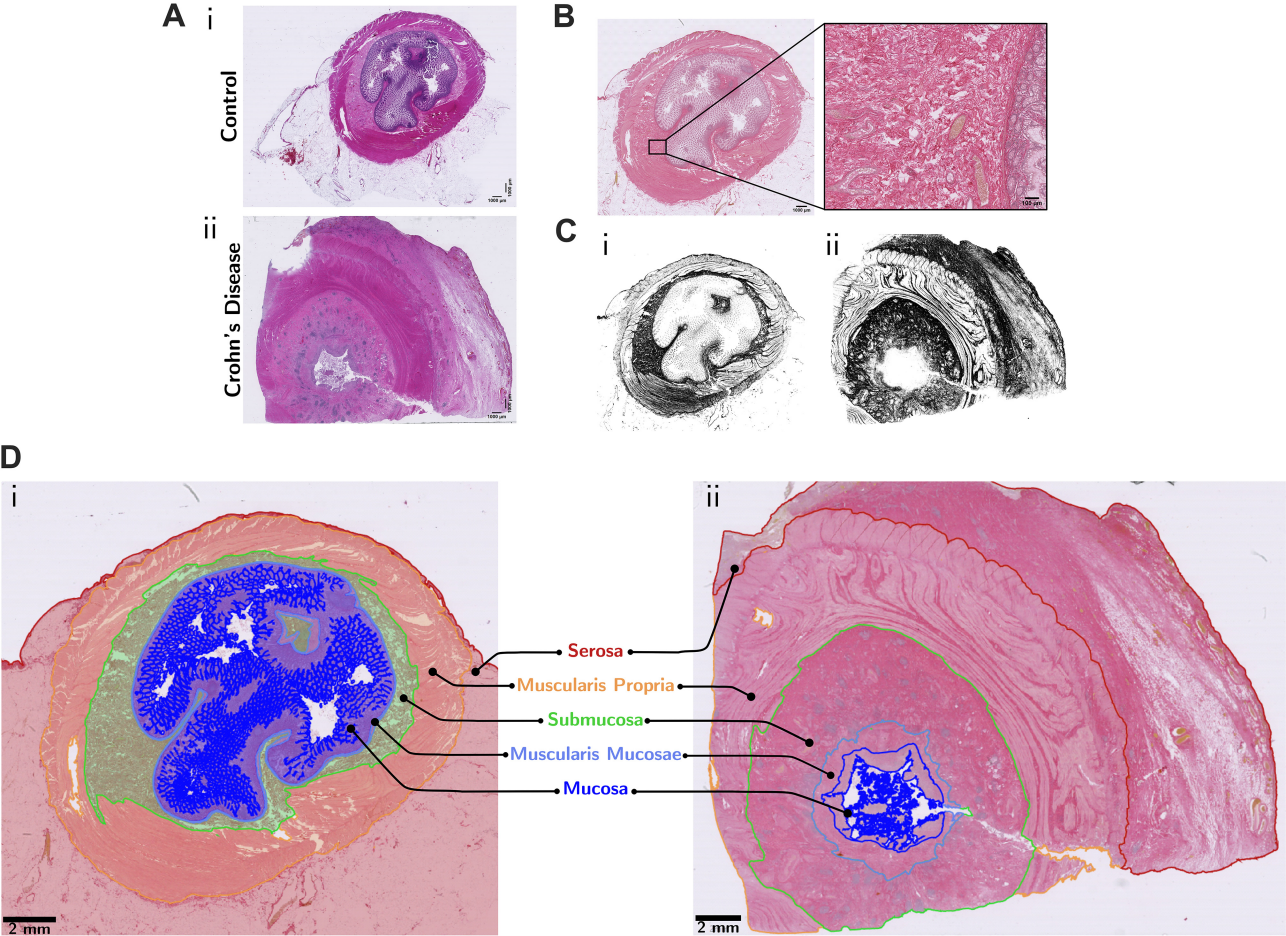
Annotations of intestinal wall layers and PSR‐stained collagen in normal and CD FSL ileum samples. (A) H&E staining of normal control (I) and CD FSL (II) samples. (B) PSR staining to visualise collagen fibres. (C) Visualisation of collagen staining based on pixel classifier output generated in QuPath for normal control (i) and CD FSL (ii) PSR‐stained samples. (D) Annotations generated in QuPath to show the layers in the ileum: mucosa (blue), muscularis mucosae (light blue), submucosa (green), muscularis propria (orange), serosa (red). Filled in annotations in normal control (i) and annotation outlines in CD FSL (ii). Scale bars, 1,000 μm in panel (A) (i) and (ii); 1,000 μm in panel (B) for whole section and 100 μm in zoom‐in; 2 mm in panel (D).

**Figure 2:**
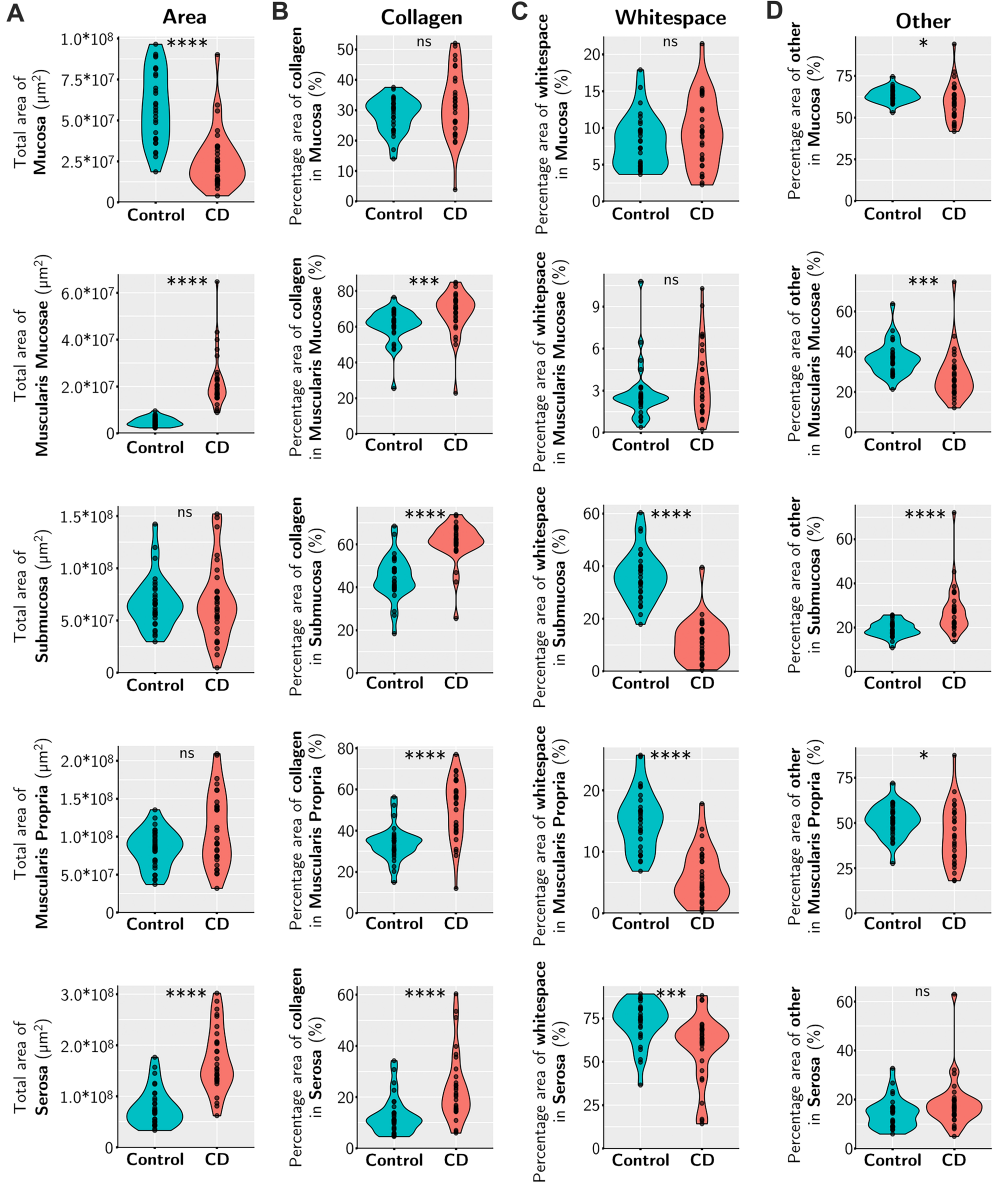
Changes in areas of intestinal wall layers and PSR‐stained collagen in normal control and CD FSL ileum samples. (A) Quantification of area for each ileal wall layer comparing normal control (blue) and CD FSL (red) samples. (B) Quantification and comparison of percentage areas of ‘collagen’, ‘whitespace’, and ‘other’ (defined in Methods) in each ileal layer. Control (blue) and CD FSLs (red). Statistical tests: non‐parametric Wilcoxon rank‐sum and signed‐rank tests: *p* > 0.05 ns, *p ≤ 0.05, **p ≤ 0.01, ***p < 0.001, ****p < 0.0001.

The main ileal layers were accurately annotated and validated by an expert GI pathologist (MJA). There was marked expansion of the muscularis mucosae, so this layer was independently annotated in addition to the four main layers (Figure 1). Layer quantification used QuPath evaluation of layer percentage areas in H&E‐stained sections of 30 normal control and 30 CD FSL samples, along with SMA IHC and PSR stains for collagen (Figure 2). To quantify collagen, a QuPath pixel classifier was trained to visualise collagen (Figure 1) and any other separate features, such as fat, immune cells, muscle tissue, vasculature, and blood cells (supplementary material, Figures S1–S3).

There was reduction in percentage area of the mucosa in CD FSL sections, due to observable widespread ulceration [40], but increased areas of muscularis mucosae and serosa (Figure 2A). A proportional increase of collagen was seen in all layers, except mucosa, in CD FSLs (Figure 2B). There was marked expansion of the muscularis mucosae layer due to increased fibrosis and smooth muscle hypertrophy/hyperplasia (Figure 2B; supplementary material, Figures S4, S9, S11).

The submucosa had a significant loss of adipose tissue whitespace together with increased collagen density in CD FSLs, compared with control ileal submucosa. There was a significant increase of ‘other’ cells in CD FSLs, which included immune cells, consistent with observed marked increases in chronic inflammation. In muscularis propria, there was increased collagen between smooth muscle fibres in CD FSLs. The serosa was expanded partly due to increased collagen and partly due to an increase in the adipose tissue whitespace consistent with observed ‘fat wrapping’ or ‘creeping fat’ (Figure 2B; supplementary material, Figures S3, S9, S11) [17, 41].

### Infiltration by B and T lymphocytes, Crohn's lymphoid aggregates (CLAs), and macrophages in CD FSL

Mucosa‐associated lymphoid tissue (MALT) represents normal clustered lymphoid follicle formation, often with germinal centres, situated mostly in the normal mucosa and sometimes extending into submucosa, most frequently seen in the terminal ileum in Peyer's patches [42]. Analysis of CD20^+^ B cells confirmed B lymphocytes present in normal ileal MALT but also showed significantly increased B lymphocytes in transmural CLAs in CD FSL (supplementary material, Figures S5–S7). CD FSLs had reduced CD20^+^ B cells in the mucosa (supplementary material, Figure S5) due to ulcerative mucosal tissue loss, but significantly increased numbers of B cells in muscularis mucosae, submucosa, and serosa layers, often in CLAs, in CD FSLs.

**Figure 3:**
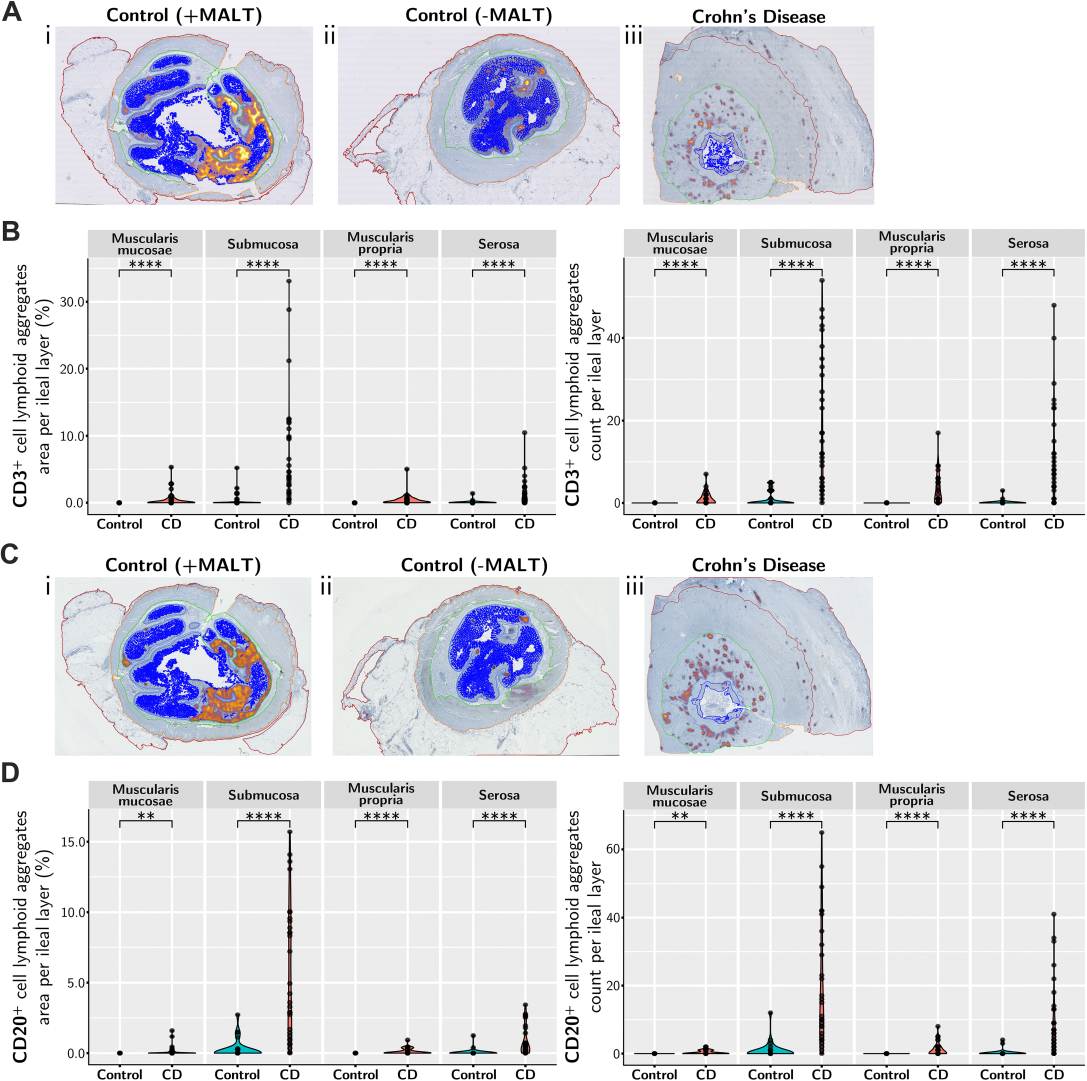
Analysis of lymphoid aggregates. Lymphoid aggregates (either CD3^+^ T‐cell lymphoid aggregates or CD20^+^ B‐cell lymphoid aggregates) were identified by QuPath density cluster map analysis. (A) Following IHC staining for CD3, QuPath density cluster map analysis showed CD3^+^ lymphoid aggregates in control ileum mucosa with MALT aggregates (I), control ileum mucosa without MALT aggregates (II), and non‐MALT‐associated CLAs in CD FSLs scattered across all ileal layers (III); the brighter the yellow/orange, the higher the T‐cell density. (B) Comparison of CD3^+^ T‐cell lymphoid aggregate accumulation per ileal layer (excluding mucosa), quantifying lymphoid aggregate percentage area normalised to region area (left) and lymphoid aggregate counts (right). (C) Following IHC staining for CD20, QuPath density cluster map analysis showed CD20^+^ B‐cell lymphoid aggregates in normal ileum mucosa with MALT aggregates (in some normal control ileum cases) (i), in control ileum mucosa without MALT aggregates (ii), and non‐MALT‐associated CD20^+^ B‐cell lymphoid aggregates in CD FSLs scattered across all ileal layers (iii). (D) Comparison of CD20^+^ B‐cell lymphoid aggregate accumulation per ileal layer (excluding mucosa), quantifying lymphoid aggregate percentage area normalised to region area (left) and lymphoid aggregate counts (right). Statistical significance for (B) and (D) from Wilcoxon rank‐sum and signed‐rank tests: *p* > 0.05 ns, *p ≤ 0.05, **p ≤ 0.01, ***p < 0.001, ****p < 0.0001.

T‐lymphocyte distribution assessed using CD3, CD4, and CD8 markers, in CD FSL compared with normal control ileum, showed significantly increased T cells in all ileal layers, except for ulcerated mucosa. Colocalisation of intermingled T and B cells was observed in transmural CLAs with some scattered lymphocytes in the mucosa, muscularis mucosae, submucosa, muscularis propria, and serosa layers (supplementary material, Figures S6 and S7). There was a striking gradient in the CD3^+^ cell distribution (supplementary material, Figure S9). Lymphoid aggregate density distribution analysis showed that in normal ileum with MALT in the mucosa, the CD3^+^ T and CD20^+^ B lymphocytes in lymphoid aggregates were in the same locations as the MALT with germinal centres (identified on H&E stains), associated with separation of B cells in germinal centres from the surrounding mantle of T cells. In contrast, in CD FSL cases, the CLAs showed colocalised and intermingled T and B cells, and these were observed in all layers of the ileum (transmural). For CD3^+^ (Figure 3; supplementary material, Figures S6 and S7) and CD20^+^ (supplementary material, Figures S5 and S7) cells, the resulting lymphoid aggregate heatmaps for normal ileum identified only MALT in the control mucosa, whereas CD FSL samples had significantly markedly increased CLAs in submucosa and serosa layers, as well as smaller increases in CLAs in muscularis mucosae and muscularis propria (Figure 3), but not in mucosa due to ulcerative loss (supplementary material, Figure S7).

Investigation of CD68^+^ macrophages showed that scattered macrophages mostly accumulated in the mucosa in normal control ileum, but are increased in number in muscularis mucosae, submucosa, muscularis propria, and serosa layers of the ileal wall in CD FSL (supplementary material, Figure S8); in particular, CD68^+^ macrophages showed a seven‐fold increase in the muscularis mucosae in CD FSL compared with normal control ileum (supplementary material, Figure S9). Only four out of 30 cases of CD FSL showed granuloma formation on macrophage cluster heatmap analysis (supplementary material, Figure S8), whereas a large majority of 26 out of 30 cases (87%), including the more severe cases of CD FSL, did not demonstrate granulomas in Crohn's fibrostenosing disease.

### Endothelial cells accumulate and form clusters around CLAs in CD FSLs

Comparison of normal control ileum with CD FSLs (supplementary material, Figure S10) showed no significant differences in CD31^+^ endothelial cell numbers between normal control mucosa and ulcerated mucosa of CD FSLs; however, there was an increase in CD31^+^ endothelial cells in the other ileal layers (supplementary material, Figure S10), with a 7.2‐fold increase in muscularis mucosae and a four‐fold increase in muscularis propria and serosa, mostly due to increased capillaries in granulation tissue or increased endothelial cells accumulating around CLAs (supplementary material, Figure S9).

**Figure 4:**
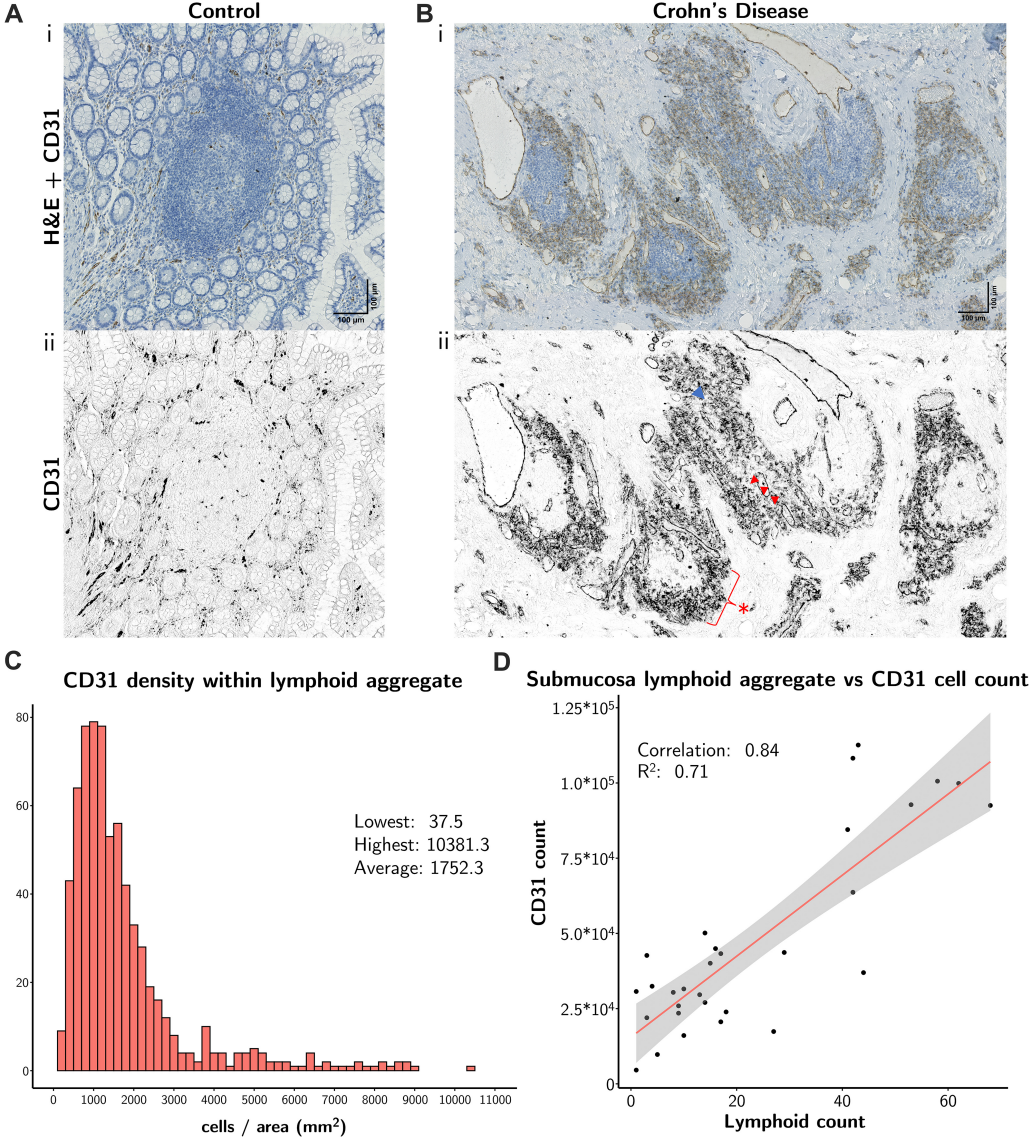
CD31^+^ cells accumulate around CLAs. IHC staining of CD31^+^ endothelial cells shows a lack of endothelial accumulation and clustering around MALT lymphoid aggregates in (A) normal control mucosa with MALT, but markedly increased numbers and clustering around CLAs in (B) CD FSL submucosa. The images show (i) H&E staining with CD31^+^ cells stained in brown and (ii) QuPath‐identified CD31^+^ cells. Blue arrowhead indicates a dilated vessel with lumen; red arrowheads indicate small capillary lumens directly adjacent to a CLA; * indicates accumulation of individual and clustered CD31^+^ endothelial cells around a CLA. (C) Histogram showing CD31^+^ cell density (raw cell number/area of CLA in mm^2^) in submucosa. (D) Scatterplot showing correlation of CD31^+^ cell count with number of CLAs (lymphoid count) present in submucosa.

The analysis showed accumulation of CD31^+^ endothelial cells, individually and in clusters, immediately surrounding CLAs present in CD FSL cases (Figure 4). In normal control mucosa, there was lack of endothelial cell accumulation around normal MALT (Figure 4), but in CD FSLs, there was an accumulation of capillaries and a large number of individual cells or clusters of CD31^+^ endothelial cells around (and some within) the CLAs (Figure 4). Endothelial cell quantification using annotations from density maps from CD20^+^ lymphoid aggregates showed that there was a dense clustering of CD31^+^ cells, with an average of 500–2,000 endothelial cells/mm^2^ around CLAs, with up to > 10,000 endothelial cells/mm^2^ (Figure 4). Further, there were no CLAs not associated with surrounding CD31^+^ endothelial cell clusters. These results were confirmed on the three fresh CD FSL samples (supplementary material, Figures S13 and S14). The CD31^+^ cell number very strongly correlated (0.84) with CLA count, with the highest correlation in submucosa (Figure 4D). Analysis of all ileal wall layers (supplementary material, Figure S12) showed good correlations between the amount of fibrotic collagen (supplementary material, Figure S11), the CLA count, and CD31^+^ cell number in submucosa and serosa and a lower correlation in the muscularis propria. Collagen amount very strongly correlated with CD31^+^ cell numbers in submucosa and serosa, whereas little or no correlation was found in other ileal layers (supplementary material, Figures S11 and S12).

### ScRNA‐seq reveals changes in cell numbers and their ligand–receptor interactions in CD FSLs

**Figure 5:**
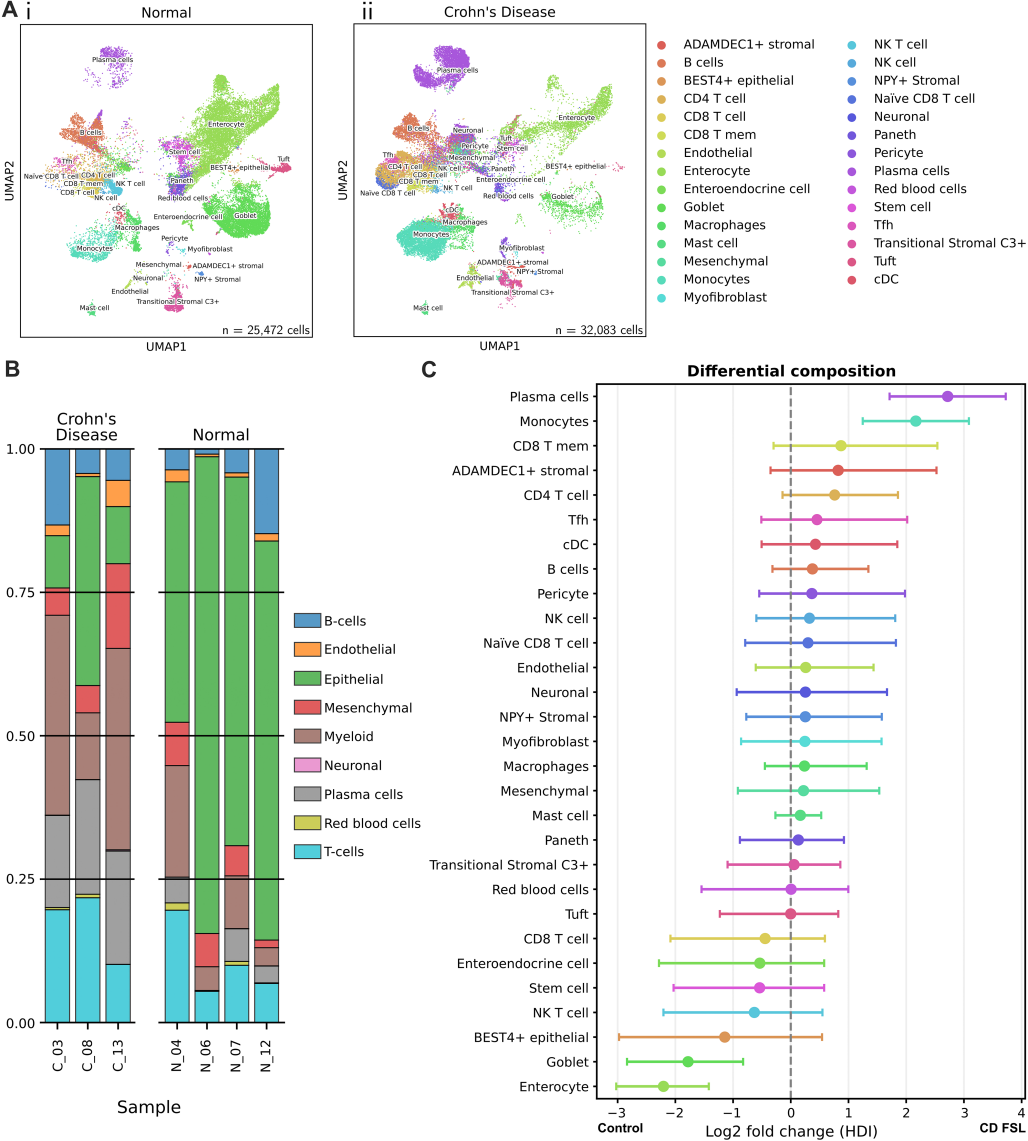
Latent representation and cell‐type compositions of scRNA‐seq data. (A) Joint UMAP embeddings of all cells coloured by cell type in (i) normal control and (ii) Crohn's disease (CD) FSL ileal samples. (B) Stacked bar graph showing distribution of cell populations within each sample and condition. (C) Log_2_ fold‐change in cell‐type composition between CD FSL ileal samples from normal controls. Points denote the posterior mean with 95% credible interval.

To investigate potential ligand–receptor signalling interactions between the relevant cell types involved in chronic inflammation and fibrosis in CD FSL, which included endothelial cells, lymphocytes from CLAs, macrophages, and mesenchymal myo/fibroblasts, we collected four normal control and three CD FSL fresh ileal samples and performed scRNA‐seq analysis on cells from transmural blocks of surgically resected whole ileal wall (Figure 5A; supplementary material, Figure S15). Annotation of cell types confirmed an increase in numbers of myeloid cells, lymphocytes, and endothelial cells, as well as inflamed epithelial cells in CD FSLs (Figure 5B), consistent with IHC‐identified cellular changes. There was a reduction in the relative proportion of the epithelial cells in CD FSLs compared with controls, reflecting mucosal ulceration, and an increase in immune and endothelial cell types (Figure 5C).

**Figure 6:**
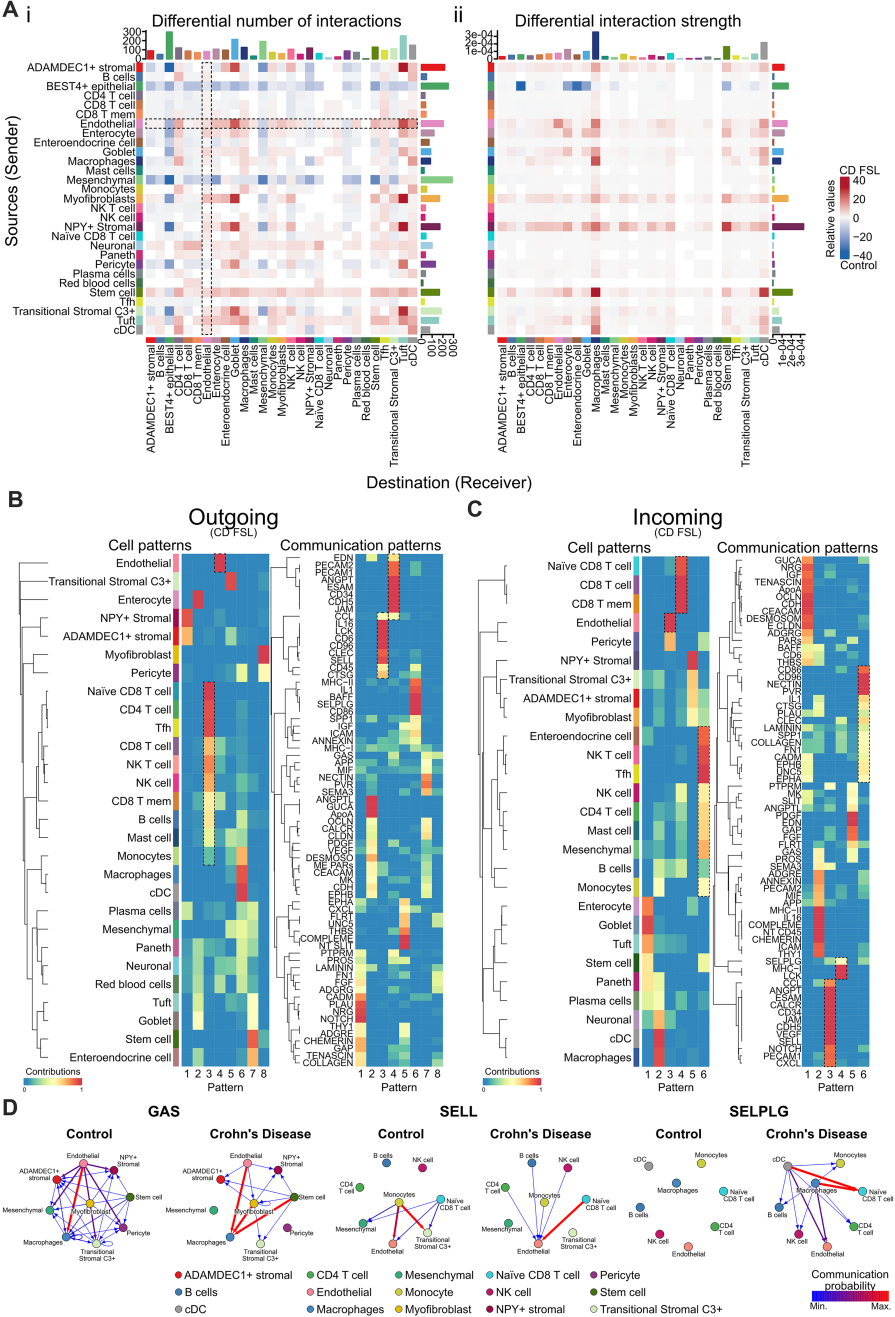
Predicted ligand–receptor signalling interactions from scRNA‐seq data using CellChat. (A) Heatmaps showing differential number of ligand–receptor interactions predicted by CellChat between curated cell types (source/sender cell on *y*‐axis and destination/receiver cell on *x*‐axis) (left), and the differential interaction strength of the associated interactions (right), comparing CD FSL annotated cells (towards red end of heat spectrum) relative to normal control annotated cells (towards blue end of heat spectrum). (B) Heatmaps showing outgoing signalling pathway patterns for each curated cell type identified in CD FSL samples (left) and the related ligand–receptor communication pathway patterns (right) for the outgoing signals. (C) Heatmaps showing incoming signalling pathway patterns for each curated cell type identified in CD FSL samples (left) and the related ligand–receptor communication pathway patterns (right) for the incoming signals. Each pattern from ‘cell patterns’ (left) corresponds to the equivalent pattern in the ‘communication patterns’ (right) heatmap, separately for outgoing signals (B) and incoming signals (C). (D) Network analysis of signalling pathways by curated cell type, showing changes in cell–cell communication pathways between normal control (left) and CD FSL (right) samples for GAS, SELL, and SELPLG signalling pathways. The arrow directions indicate the communication pathway's direction and the arrow colour indicates the strength of the communication probability (blue indicating low/minimum and red indicating high/maximum).

Intercellular communication networks between cell types were inferred from expression of known ligand–receptor pairs using CellChat (Figure 6; supplementary material, Figure S16), especially in immune and endothelial cell types to compare signalling behaviour between CD FSLs and normal controls (Figure 6; supplementary material, Figure S17–S19). Higher‐order communication networks were identified from groups of pathways and cell types sharing similar signalling profiles, with the optimum number of patterns for each condition determined from silhouette and cophenetic correlation metrics based on the elbow heuristic. Specific signalling pathway patterns were identified (Figure 6) in CD FSLs for specific cell types, such as PECAM1 and PECAM2, ANGPT, ESAM, CD34, CDH5, and JAM were observed as the highest contributors to Pattern 4 of outgoing signals from endothelial cells; whereas for B, T, and NK cells’ outgoing signals, Pattern 3 signalling pathways included IL16, LCK, CD6, CD96, CLEC, SELL, and CD45. Interestingly, Patterns 4 and 6 for incoming signals for mesenchymal cells and lymphocytes included signalling pathways such as CD86, CD96, NECTIN, PVR, IL1, CLEC, COLLAGEN, FN1, SELPLG, MHC‐I, and LCK, among others (Figure 6). In general, endothelial cells had the most active outgoing and incoming signalling pathways (Figure 6; supplementary material, Figures S16–S19).

Network analysis of signalling pathways between cell pairs that differed in CD FSLs compared with normal controls identified several prominent pathways, including GAS (endothelium, stromal cells, and stem cells signalling to macrophages), SELL (CD8 T cells signalling to endothelium), and SELPLG (macrophages and dendritic cells signalling to CD8 T cells and endothelial cells) (Figure 6), and additionally other less prominent signalling interactions involving ANNEXIN, CD45, CCL, CXCL, COLLAGEN, EDN, FGF, FLRT, FN1, ICAM, IL1, IL16, MHC‐I, NECTIN, NOTCH, PDGF, PECAM1/2, PROS, THBS, THY1, and VEGF pathways between endothelial cells, T cells, B cells, macrophages, and myofibroblasts (supplementary material, Figure S20).

Collectively, these observations provide some support for the presence of multiple ligand–receptor signalling pathways involved in intercellular communication between pathologically accumulated endothelial clusters around lymphoid aggregates with T and B lymphocytes, macrophages, and myofibroblasts that may be involved in the formation of fibrotic collagen in CD FSLs, but this requires further investigation and validation.

## Discussion

In this study we have comprehensively characterised and quantified cellular, smooth muscle, and fibrotic collagenous changes present in the ileal wall layers of surgically resected ileal CD FSLs relative to normal controls. These changes included increased fibrotic collagen in almost all layers of the ileum, most marked in the submucosa, a 4.5‐fold expansion of the muscularis mucosae due to increased collagen deposition and smooth muscle hyperplasia/hypertrophy, along with serosal expansion predominantly caused by fat wrapping with increased fibrotic collagen, which has been insufficiently emphasised in previous histopathological descriptions of CD FSL [43].

The data quantify the layer‐specific amounts of collagen deposition in FSLs, showing a wider‐than‐expected range of collagen deposition, affecting almost all layers of the ileal wall (muscularis mucosae, submucosa, muscularis propria, and serosa), which have not been described in such detail heretofore. SMA immunostaining allowed quantification of the prominent smooth muscle hyperplasia/hypertrophy in CD FSLs, most notably observed in the muscularis mucosae and the muscularis propria, with muscularis mucosae expanded in CD FSLs, sometimes to the point of connecting the smooth muscle fibres of the muscularis mucosae to those of the expanded muscularis propria in some cases [5]. This smooth muscle hyperplasia/hypertrophy could be explained by cytokine release from infiltrating lymphocytes and macrophages, along with fibroblast activation and formation of myofibroblasts contributing to fibrosis [5, 10, 13]. Activation of myofibroblasts was identified by SMA immunostaining, also presumably as a result of infiltration by immune cells throughout the ileal layers. The partial luminal obstruction in CD FSLs is due to expansion of almost all ileal wall layers in particular muscularis mucosae, submucosa, and muscularis propria with a reduction of the lumen [44]. The most marked fibrotic collagenous changes occur in submucosa, suggesting that the submucosa layer may be the nucleation focus for the process of fibrosis, which spreads to other adjacent layers, and this is consistent with this submucosa layer becoming more ‘rigid’, creating a barrier ensuring that muscularis mucosae expansion occurs inwards more towards the lumen.

The process of chronic inflammation and subsequent fibrosis has been linked to cytokines released from immune cells. Interleukins and other cytokines released by dendritic and other cells can activate T cells (CD4 T cells and Treg cells [45, 46, 47, 48, 49]), leading to trans‐differentiation of CD4 T cells into CD4 Treg cells. The CD4 Treg cells can release many cytokines that may activate fibroblasts and myofibroblasts [4, 6, 50]. Our findings show that there is a significant increase of CD3^+^, CD4^+^, and CD8^+^ T lymphocytes in most layers of the ileum, except ulcerated mucosa, with the most marked increase in CLAs in the submucosa. Previous studies showed an increase in CD3^+^ [51, 52], CD4^+^, and CD8^+^ T cells [51] present in CD mucosa and some superficial submucosa (as biopsy studies have limited depth of gut wall tissue accessible for analysis), which correlates with our findings for mucosa and submucosa, but these biopsy studies were unable to show cellular changes in deep submucosa, muscularis propria, and serosa. Furthermore, analysis of CD20^+^ B‐cell distribution confirmed the accumulation within germinal centres of MALT separated from surrounding mantle T cells in mucosa in normal controls, in contrast to the intimately mixed B cells and T cells in CLAs in all layers [53]. Quantification of transmural CLAs in CD FSLs showed the largest increase in B‐ and T‐cell‐containing CLAs in the submucosa, followed by the serosa of CD FSL, confirming their diagnostic utility in CD and raising the possibility of a relationship with fibrosis, as most CLAs correlated with most collagen in the submucosa layer. The CLAs have a different internal structure with close colocalisation of B lymphocytes and T lymphocytes. CLAs were found throughout the layers of the ileal wall (except mucosa) mostly in scattered single aggregates, whereas MALTs were exclusively localised in mucosa and were often confluent creating large clusters of lymphoid follicles. Therefore, the presence of CLAs in other layers cannot be explained by simple migration of MALT aggregates towards other layers of the ileum [54].

Most of the cases of CD FSLs (26 out of 30, 87%), including the more severe cases of CD FSLs, did not have granulomas present, supporting the interpretation that the presence of granulomas is not required for the development of Crohn's fibrostenosing disease. However, widespread, scattered individual CD68+ macrophages are present in all CD FSL cases, consistent with an important role in the disease mechanisms of chronic inflammation and fibrosis [55, 56, 57, 58]. As mentioned earlier, the release of cytokines leads to the activation of various cell types, and these include activation of macrophage differentiation into M2 macrophages, which are known to be profibrotic [59]. The presence of several cytokines can lead to the activation of epithelial‐to‐mesenchymal transition processes, stimulating increases of the mesenchymal‐like cell pool [10, 11]. The activated fibroblasts and myofibroblasts lay down a collagen‐rich matrix, which is involved in a chronic build‐up of fibrosis, eventually leading to the formation of a FSL and obstruction of the lumen. Our findings showed a significant increase in cell numbers for CD68^+^ macrophages (as well as the SMA^+^ expansion). A similar result was induced in rats, where an artificially induced colitis was characterised by the presence of CD68^+^ macrophages, presence of hypoxia‐inducible factor (HIF)‐1α, and expansion of muscle layers [60]. In fact, the hypoxic environment present around the fibrotic tissue, involving hypoxia‐inducible factor (HIF)‐1α, further enhances polarisation of macrophages towards profibrotic M2 macrophages [10, 11, 61, 62, 63], with the expression of neoangiogenic genes such as vascular endothelial growth factor (VEGF), contributing to granulation tissue formation in CD [64, 65, 66]. This can be indirectly observed from the overall increased numbers of endothelial cells in CD FSLs, consistent with stimulation by VEGF and other mediators and their accumulation with involvement in the formation of granulation tissue capillaries with surrounding fibrotic collagen deposition by fibroblasts.

Central to FSL development in CD, the most pathologically significant finding was that the greatest accumulation of collagen was found in the submucosa – which we hypothesise to be a ‘nucleation’ focus, where the initial stages of inflammation‐driven fibrosis begin and progress through infiltration of immune cells, both lymphocytes and macrophages, leading to release of cytokines that stimulate the fibrosis via signalling pathways with the expansion and activation of fibroblast and myofibroblast populations [23]. The colocalisation and correlation of the densest accumulation of endothelial‐CLAs and the highest levels of fibrosis in the submucosal layer is consistent with CLAs playing a role in the formation of collagenous fibrosis in CD, but this requires further investigation, such as by spatial transcriptomic analysis. Increased endothelial cells in CD were previously described [65], but here we demonstrate with quantification that there is marked endothelial cell accumulation in single cells, mostly clusters, and some capillary vessels surrounding the CLAs in submucosa and other layers. We hypothesise that the combination of endothelial cells and B and T lymphocytes in these CLAs interact with each other through ligand–receptor interactions via signalling pathways including CD86 (regulation of lymphocyte migration [67]), SELL (L‐selectin) (recruitment of immune cells [68]), ANGPT (stimulator of pro‐inflammatory and pathogenic endothelial cells) [69], GAS (involved in promoting fibrosis [70]), and SELPLG (involved in promoting tumour formation [71]) in the formation of these endothelial–lymphocyte aggregates (Figure 6; supplementary material, Figures S15–S20). The combination of B and T lymphocytes with endothelial cells interact with fibroblasts/myofibroblasts either directly or indirectly via macrophages, through multiple ligand–receptor signalling pathways, identified by the scRNA‐seq analysis, including pathways such as GAS, SELL, SELPLG, COLLAGEN, FGF, NECTIN, and PDGF, among others, for which there is evidence to support roles in modulating fibrosis, and thus we hypothesise that combinations of these signalling pathways can stimulate pathological myofibroblast activation and increased synthesis of collagen and matrix glycosaminoglycans, resulting in increased collagenous fibrosis [72, 73, 74, 75, 76, 77]. In liver fibrosis, interactions between distinct macrophages and endothelial cell subsets were found to promote hepatic stellate cell activation in the fibrotic liver, consistent with a conserved mechanism of fibrosis across organs [78].

There are some limitations to our study. Although we were successful in clarifying the location of major cell types in each ileal layer, lack of specific cell surface markers for identification of fibroblasts or myofibroblasts limited their investigation by IHC. Some previous studies attempted to identify fibroblasts in CD through a combination of vimentin, fibroblast activation protein (FAP), and/or SMA, but those cover a large number of mesenchymal cells, and FAP immunostaining resulted in non‐specific staining of many cell types [79]. Furthermore, the issue of heterogeneity of fibroblast and myofibroblast populations complicates IHC antibody analysis. A number of studies have identified potentially unique fibroblast markers such as KIAA1199 [80] or cadherin‐11 (CDH11) [81]. The study by Mukherjee *et al* [81] on CDH11 suggested that CDH11 may be a potential target for treating fibrosis as blocking the expression of the protein led to reduction of ECM deposition. Nevertheless, further investigation of CD FSL fibroblast and myofibroblast populations with the use of spatial transcriptomics might identify more unique cell surface markers and provide improved insights into this process of FSL development and progression. In summary, our study shows the highest levels of fibrotic collagen and accumulated endothelium‐CLAs in submucosa, consistent with CLAs potentially having a role in promoting fibrosis in CD FSLs, representing a novel mechanism for anti‐fibrosis drug‐targeting, albeit requiring further investigation. These data provide the much‐needed benchmark spatially quantifying the cellular and molecular architecture of CD FSLs, and the data add further evidence that Crohn's fibrotic tissue is not inert scar tissue, as previously thought, but is a vascularised dynamic tissue that may be amenable to regression with targeted immune modulation and/or antifibrotic therapy.

## Author contributions statement

All authors participated in manuscript preparation and review. MG was responsible for dissociation and preparation of fresh ileal samples, staining and image acquisition, QuPath analysis and analysis of data. FN and GJW performed scRNA‐seq analysis. SD identified and obtained consent from participants. KJK acquired archival and fresh ileal samples. HC undertook histological processing and sectioning. MW, BHill, BHaggarty, DH, MS, FN, AK, SD and MJA contributed to data analysis and provided manuscript draft support. DJA performed sequencing. AB, RAB, DJA, IP, SD, PB and MJA provided PI and analysis support. PB performed QuPath coding. MJA provided leadership, strategic and experimental support and supervision of the histopathological and immunohistochemical analysis.

## Supporting information


Supplementary materials and methods
**Figure S1.** Exported collagen pixel classifier images for normal control ileal samples
**Figure S2.** Exported collagen pixel classifier images for Crohn's disease ileal fibrostenosing lesion samples
**Figure S3.** Fat wrapping or creeping fat formation in serosa of Crohn's disease FSL samples
**Figure S4.** SMA^+^ immunohistochemical staining analysis of smooth muscle cells in normal control and CD FSL samples
**Figure S5.** Quantification and distribution of B‐lymphocytes
**Figure S6.** Quantification and distribution of T‐lymphocytes
**Figure S7.** Lymphoid aggregate quantification in mucosa
**Figure S8.** CD68^+^ macrophage quantification and granuloma identification
**Figure S9.** Bar chart of mean cell number fold‐change
**Figure S10.** CD31^+^ endothelial cell population analysis
**Figure S11.** Bar chart of mean fold‐change for collagen (%), whitespace (%), other (%), and layer areas (μm^2^)
**Figure S12.** Correlation analysis between CLA counts, CD31+ cell counts, and amount of collagen (μm^2^)
**Figure S13.** H&E, PSR, and immunohistochemical stained photomicrograph images of four freshly collected normal control ileum and three freshly collected CD FSL samples used in scRNA‐seq analysis
**Figure S14.** Photomicrograph images of immunohistochemical stains for CD31+ endothelial cells showing a marked increase and accumulation of CD31+ cells around CLAs in freshly collected CD FSL samples used in scRNA‐seq analysis
**Figure S15.** Overview of scRNA‐seq data with table of QC metrics, UMAP visualisations, compositional model coefficients, and marker genes of four normal control and three Crohn's disease FSL ileum samples
**Figure S16.** Analysis of ligand–receptor signalling interactions between pairs of cell types using CellChat
**Figure S17.** Higher‐order signalling patterns between cells in normal control sample data
**Figure S18.** Higher‐order signalling patterns between cells in CD FSL sample data
**Figure S19.** Ligand–receptor signalling interaction patterns between cell type categories in normal control and CD FSL sample data
**Figure S20.** Pathway communication networks between curated cell types or cell type categories in normal control and CD FSL sample data
**Table S1.** Archival sample details
**Table S2.** Immunohistochemistry conditions for specific antibodies
**Table S3.** Mucosa correction pixel classifier settings
**Table S4.** Collagen detection pixel classifier settings
**Table S5.** Positive cell detection settings for each antibody
**Table S6.** Density map function settings for CD68+ cell granulomas and CD3+ and CD20+ lymphoid aggregates
**Table S7.** Density map threshold settings

## Data Availability

Images used for the analysis can be found here: https://workbench-czi-cpw.mvm.ed.ac.uk/public_matrix/69/. The archival image data (annotations and extracted quantified data) as well as the code are available upon request. The scRNA‐seq data may be available upon request.
