# Supplementary material for: Crohn's lymphoid aggregates with endothelial clusters colocalise with submucosal fibrosis in fibrostenosing Crohn's disease
Source: J Pathol. 2026 Feb 5;268(4):398–412. doi: 10.1002/path.70019 (PMC12984004; doi:10.1002/path.70019)
Supplement: Supplementary file 1 — Supplementary materials and methods Figure S1. Exported collagen pixel classifier images for normal control ileal samples Figure S2. Exported collagen pixel classifier images for Crohn's disease ileal fibrostenosing lesion samples Figure S3. Fat wrapping or creeping fat formation in serosa of Crohn's disease FSL samples Figure S4. SMA+ immunohistochemical staining analysis of smooth muscle cells in normal control and CD FSL samples Figure S5. Quantification and distribution of B‐lymphocytes Figure S6. Quantification and distribution of T‐lymphocytes Figure S7. Lymphoid aggregate quantification in mucosa Figure S8. CD68+ macrophage quantification and granuloma identification Figure S9. Bar chart of mean cell number fold‐change Figure S10. CD31+ endothelial cell population analysis Figure S11. Bar chart of mean fold‐change for collagen (%), whitespace (%), other (%), and layer areas (μm2) Figure S12. Correlation analysis between CLA counts, CD31+ cell counts, and amount of collagen (μm2) Figure S13. H&E, PSR, and immunohistochemical stained photomicrograph images of four freshly collected normal control ileum and three freshly collected CD FSL samples used in scRNA‐seq analysis Figure S14. Photomicrograph images of immunohistochemical stains for CD31+ endothelial cells showing a marked increase and accumulation of CD31+ cells around CLAs in freshly collected CD FSL samples used in scRNA‐seq analysis Figure S15. Overview of scRNA‐seq data with table of QC metrics, UMAP visualisations, compositional model coefficients, and marker genes of four normal control and three Crohn's disease FSL ileum samples Figure S16. Analysis of ligand–receptor signalling interactions between pairs of cell types using CellChat Figure S17. Higher‐order signalling patterns between cells in normal control sample data Figure S18. Higher‐order signalling patterns between cells in CD FSL sample data Figure S19. Ligand–receptor signalling interaction patterns between cell type categories in normal [file PATH-268-398-s001.docx]

**Crohn’s lymphoid aggregates with endothelial clusters colocalize with submucosal fibrosis in fibrostenosing Crohn’s disease**

M Glinka, GJ Wickham, F Nadalin *et al. J Pathol* <https://doi.org/10.1002/path.70019>

**Supplementary materials and methods**

**Supplementary Figures S1–S20**

**Supplementary Tables S1–S7**

# Supplementary materials and methods

## Tissue acquisition and processing

### Archival sample acquisition

The archival 30 normal control ileum (control, additional normal tissue from unrelated conditions that do not present any ileal abnormalities) and 30 CD FSL samples from ileum were acquired from NHS Bioresource under ethics approval 20/ES/0061 (SR1492) (supplementary material, Table S1). The tissue had been fixed in formalin for 24–48 h and then processed and embedded in paraffin. In brief, the tissue was dehydrated in increasing ethanol concentrations (50%, 75%, 100%), followed by clearing with xylene and embedding in paraffin wax. The tissue was sectioned on a microtome at 4 µm thickness.

### Fresh sample acquisition and dissociation into single-cell suspensions

The four fresh normal control ileum samples (control, additional normal ileal tissue from unrelated conditions that do not present any ileal abnormalities) and three CD FSL samples from ileum were acquired from surgical resection specimens from prior consenting patients under the NHS Bioresource ethics approval 20/ES/0061 (SR1803). The tissue was examined, dissected, processed, and dissociated fresh (within 30 min of acquisition) to acquire single-cell suspensions based on previously published protocols [36] with slight modifications. A fresh ileal sample was sectioned by a pathologist into two separate adjacent tissue rings – one for fixation for histopathological analysis and the other for fresh dissociation into single cells for scRNA-seq analysis. The sample for histopathological analysis was directly submerged in 10% formalin for 24 h fixation, whereas the sample for dissociation was kept fresh and stored in ice-cold HBSS^−/−^ buffer (HBSS without Ca^2+^ and Mg^2+^, 14175053, Gibco, Life Technologies, Glasgow, UK) and was immediately taken for processing. The preparation steps involved setting up the centrifuge to 4 °C and storing all reagents on ice. Prior to digestion, digest buffers 1 and 2 were prepared as follows: digest buffer 1 was prepared with 2 mM EDTA (51201, Lonza, Scientific Laboratory Supplies Ltd., Lanarkshire, UK) in HBSS^−/−^ buffer (diluted 0.2 ml of 0.5 M EDTA in 49.8 ml of HBSS^−/−^); digest buffer 2 was prepared with 5 mM CaCl_2_ (21115-100 ml, Sigma/Merck, Dorset, UK) by diluting 250 µl of 1 M CaCl_2_ in 49.75 ml of HBSS^+/+^ (HBSS with Ca^2+^ and Mg^2+^, BE10-527F, Lonza, Scientific Laboratory Supplies Ltd.). Once the sample was acquired, the following were added: 1,800 µl of digest buffer 1, 10 µl solution (in water) of 10 mM Y-27632 dihydrochloride Rho kinase inhibitor (ROCK-I) (ab120129-10 mg, Abcam, Cambridge, UK), 10 µl solution (in DMSO) of 1 mM QVD-OPh (caspase-3,1,8, and 9 inhibitor, ab141421-1 mg, Abcam), and 200 µl solution (in HBSS^−/−^) of 1,300 U/ml Bacillus Licheniformis Protease (BLP, P5380-250 mg, Sigma/Merck). The tissue sample was then macerated by holding the tissue with forceps and shredding it with a scalpel in a glass Petri dish. Once the tissue was minced, it was then transferred to a tube with digest buffer solution 1 for 30 min, with gentle mixing every 10 min. After 30 min, 800 µl of 25% BSA (A9543-5g, Sigma/Merck) solution (in HBSS^−/−^) was added to quench the enzymes, and the tube was topped up to 10 ml using ice-cold HBSS^−/−^. The tissue suspension was then centrifuged at 350 × *g* for 10 min, and the supernatant was removed. Digest buffer solution 2 was added to the same tube as 1,680 µl of digest buffer 2, 200 µl of Dnase I (07900, Stem Cell Technologies, Cambridge, UK), 60 µl solution (in HBSS^+/+^) of 50 U/µl collagenase IV (17104019 1 g, 225 U/mg, Life Technologies, Glasgow, UK), and 60 µl solution (in HBSS^+/+^) of 300 U/ml hyaluronidase (H1136-1AMP, Sigma/Merck), and the sample was left to incubate at 37 °C for 15–20 min. Once the digestion was complete, 20 µl of 0.5 M EDTA was added, and the sample was strained through a 70-µm-cell strainer (352350, Falcon, Scientific Laboratory Supplies Ltd., Lanarkshire, UK), followed by a 40-µm-cell strainer (352340, Falcon, Scientific Laboratory Supplies Ltd.), while washing down any cells with 1% BSA in HBSS^−/−^ wash buffer. The sample was then centrifuged at 350 × *g* for 10 min and treated with 2 ml ACK lysis buffer (A10492-01, Gibco, Life Technologies) to remove any red blood cells, followed by topping up with wash buffer and centrifugation at 350 × *g* for 5 min. The supernatant was removed, and the cells resuspended with a wash step with a 30-µm-cell strainer (04-0042-2316, CellTrics, Wolflabs, York, UK) was performed. The sample was then centrifuged again at 350 × *g* for 5 min and resuspended in a minimal amount of wash buffer for cell counting using Trypan Blue (15250061, Sigma/Merck) and counted on a haemocytometer (DHC-N01, NanoEnTek, Cambridge BioScience, Cambridge, UK). If any debris or dead cells were still present, a wash step was performed followed by centrifugation at 350 × *g* for 5 min and recounting the cells.

## H&E staining

The sectioned tissue was rehydrated in a stepwise manner, starting with xylene to remove the paraffin, then in alcohol solutions with decreasing concentrations (100% ×2, 80%, 50%) followed by washing with water after each step, staining with haematoxylin (10694409, Fisher Scientific, Leicestershire, UK) for 5 min, blueing with Scott’s Tap Water Solution [STWS, pre-made mixture of sodium bicarbonate (S5761, Sigma/Merck) and magnesium sulphate (CHE2456, SLS, Lanarkshire, UK)] for 1 min and staining with eosin (10188418, Fisher Scientific) for 5 min. The sample was then dehydrated with the reverse of the above steps. The samples were then mounted in mounting media [Pertex (SEA-0100-00A, Cellpath, Powys, UK)].

## Picrosirius Red (PSR) staining

Sections were rehydrated as above and stained with haematoxylin for 5 min and blued with STWS for 2 min. The next step involved encircling the sections with a hydrophobic barrier pen and staining the sections with PSR (ab150681, Abcam) solution for 8 min in the dark. The slides were washed for at least 5 min for the colour to fully develop and then dehydrated as above.

## IHC staining and visualisation of positive cells

To visualise specific cell types, surface marker antibodies were utilised in conjunction with DAB oxidisation to produce brown stain for positive cells. The sections were dehydrated as stated above, followed by blocking of endogenous peroxidase activity with hydrogen peroxide at a final concentration of 3% for 20 min. The antigens present on the surface of the cells were retrieved using Tris-EDTA buffer (10 mM Tris base, 1 mM EDTA solution, 0.05% Tween 20, pH 9.0) under high pressure in a pressure cooker (Microwave Tender Cooker 2.5-Quart, NordicWare, Minneapolis, MN, USA) (see supplementary material, Table S2, for specific conditions for each antibody). The sections were allowed to cool and permeabilised using 0.5% Triton-100X solution in TBS for 20 min. The sample area was encircled using hydrophobic barrier pen and blocking of any non-specific antigen binding was performed using 5% goat serum in 0.1% Tween-20 solution in TBS for 1 h. Following the blocking step, primary antibody was added for overnight incubation at 4 ^o^C, followed by washing steps and secondary antibody staining the next day for 2 h at room temperature. The DAB solution (K346811-2, Agilent, Cheadle, UK) was prepared as instructed (one drop of DAB concentrate in 1 ml of peroxidase buffer) and applied to the sections for a maximum of 2 min (see supplementary material, Table S2, for details) and washed in water to remove excess solution. The sections were stained with haematoxylin and STWS as described above, followed by dehydration as above and mounting.

## Whole-slide scanning imaging

The stained tissue sections were scanned using the Hamamatsu Nanozoomer XR Slidescanner (Welwyn Garden City, UK).

## QuPath image analysis

All images were analysed with QuPath 0.5.1 [35] using relevant actions in QuPath as described (<https://qupath.readthedocs.io/en/0.5/> and <https://github.com/qupath/qupath-extension-omero-web>).

### Annotation of intestinal wall layers

For each stain a new project folder was created within QuPath. Each ileal wall layer (mucosa, muscularis mucosae, submucosa, muscularis propria, and serosa) was manually annotated [Figure 1C (i) and (ii)]. Each stain vector was then corrected semi-automatically in QuPath (Analyze > Estimate stain vectors). To correct the mucosal layer for removal of the lumen (and its contents) that was present in the original annotation of the mucosa, a new pixel classifier was trained to detect all tissue except for the lumen and then applied to the existing mucosa annotations to ensure correct identification of the mucosal tissue layer without luminal space. The tissue regions were selected and marked as ‘mucosa’ and the lumen as ‘ignore’.

The specific options chosen for the pixel classifier (Classify > Pixel classification > Train pixel classifier) are described in the supplementary material, Table S3.

Once the QuPath pixel classifier had been trained, it was used to generate more detailed annotations for the ‘mucosa’ class. Following correction for luminal space, the original manual mucosa annotation was deleted, but the corrected descendant mucosa annotation was kept. Small fragments (less than 6,000 µm^2^) were removed, and small holes (less than 1,500 µm^2^) were filled (Objects > Annotations… > Remove fragments and holes). Finally, the annotations were simplified to improve performance by reducing the number of vertices while preserving the main shape (Objects > Annotations… > Simplify shape, with altitude threshold 30).

### Collagen quantification

To quantify the amount of collagen present in the different layers of intestinal tissue stained with PSR, a QuPath pixel classifier was trained to detect ‘collagen’ (Collagen, pink), ‘whitespace’ (to include lipid droplets in fat cells, vascular lumen, and white background) (Negative, blue), and ‘other’ (to include infiltrating inflammatory or immune cells, red blood cells, other cells, and smooth muscle tissue) (Other, yellow). Each stain vector was corrected semi-automatically (Analyze > Estimate stain vectors).

The training of the classifier was performed on randomly selected 500 × 500 px boxes (total of 52) encompassing all ileal layers, from six total (three normal, three  CD FSL) sections. QuPath allows for generation of a training image that combines all the randomly selected boxes into one large image, which in turn is then annotated in as detailed a manner as possible for ‘collagen’, ‘whitespace’, and ‘other’. The resulting classifier was then visually checked by histological examination by a specialist GI pathologist (Mark Arends) on both the training dataset as well as actual full-size images of sections chosen at random to confirm the validity of the classification. If the result was unsatisfactory, the annotations were repeated on the training images. The resulting pixel classifiers are available at GitHub as a .zip file: https://github.com/Comparative-Pathology/GCA_QuPathLayerAnnotations

This was validated by histological examination by a specialist GI pathologist (Mark Arends) to confirm its accuracy. The specific settings used for training the pixel classifier are described in the supplementary material.

The pixel classifier was then applied to all the images using batch scripting (Automate > Script editor) and the generated images (supplementary material, Figures S1 and S2), and quantified data were exported and analysed using Python and R. The collagen, whitespace, and other raw area values (µm^2^) were normalised to the total area of the specific region as per the formula below:

$$Percentage area \left( \% \right)= \frac{Area of object of interest ({\mu m}^{2})}{Area of the ileal region ({\mu m}^{2})}\times100$$

### Quantification of immunohistochemically identified DAB-positive cells

The immunohistochemically identified DAB-positive cells stained in brown were quantified using the built-in ‘Positive cell detection’ function (Analyze > Cell detection > Positive cell detection) of QuPath. For each antibody, a stain vector was corrected using the auto function (Analyze > Estimate stain vectors) in QuPath. Supplementary material, Table S5, contains the specific settings used for positive cell detection for each antibody.

The resulting data were then extracted using batch scripting and analysed using Python and R.

### Density map analysis for granulomas and lymphoid aggregates

For granuloma and lymphoid aggregate detection, the ‘Density Map’ (Analyze > Density Maps > Create Density Maps) function in QuPath was used. The function maps clusters of the positively stained cells according to their density and through application of thresholds allows for identification of cell aggregations, including granulomas (CD68+ macrophage aggregates) and lymphoid aggregates (either CD3+ T-cell aggregates or CD20+ B-cell aggregates). Supplementary material, Table S6, shows the settings used for setting up the density maps for quantifying cell aggregates by percentage area or aggregate counts.

The density map was saved and re-applied to all images (Analyze > Density Maps > Load Density Map) based on the most optimal setting as shown in the supplementary material, Table S7.

The resulting annotation area was recorded and exported to ImageJ where a mask was generated (Right click on image > Create Mask). The resulting image was analysed by quantifying the particles (Analyze > Analyze Particles) with the following settings: Size (µm^2^): 20,000 – infinity, Show: outlines. The data for each image were collated and further analysed using Python and R.

## CD31^+^ endothelial cell proximity to lymphoid aggregates analysis

The density maps generated for CD20^+^ cell clusters were used as annotations on the CD31^+^ IHC images and manually corrected to match the position of lymphoid aggregates. The positive cell detection (as described in 0) was used to quantify the CD31^+^ cells within the lymphoid aggregates. The cell density was then calculated by dividing the cell number in each particular lymphoid aggregate over the area of said lymphoid aggregate and visualised in the form of a histogram to show the distribution.

## Computational analysis with Python and R

The data from QuPath were collated using Python 3 script into .csv files. The results were analysed using R (R-4.3.3) in RStudio (RStudio 2023.12.1 Build 402) with the following libraries: tidyverse (2.0.0), dplyr (1.1.4), tidyr (1.3.1), ggplot2 (3.5.0), rstatix (0.7.2), ggpubr (0.6.0), ggsignif (0.6.4), gridExtra (2.3), broom (1.0.5), purr (1.0.2), svglite (2.1.3), moments (0.14.1). All code for the analysis is available on GitHub: <https://github.com/Comparative-Pathology/GCA_QuPathLayerAnnotations>.

## Mean fold-change

The arithmetic mean was calculated for all 30 data points for normal control and CD samples based on the following formula:

$$\bar{x}= \frac{1}{n}\left( \sum_{i=1}^{n} x_{i} \right)=\frac{x_{1}+x_{2}+\cdots+x_{n}}{n}$$

Once the mean was calculated, a mean fold-change was derived from dividing the CD sample mean by normal control sample mean:

$$Mean fold-change= \frac{\bar{x}_{Crohn^{'}s Disease}}{\bar{x}_{Control}}$$

## scRNA-seq library generation and data analysis

We set out an analysis workflow aimed at accurately annotating cell types using a combination of semi-supervised and unsupervised approaches. First, we leveraged the curated annotation from publicly available data [37] to automatically label cells from ileum samples based on gene expression similarity (semi-supervised); second, we computed cell clusters and obtained a curated annotation using information from both the automatically assigned labels and the cluster markers.

Given the high number of loaded cells potentially resulting in a doublet rate equal or greater than 7.6% (following the manufacturer’s protocol; 10X Genomics, Pleasanton, CA, USA), we performed a thorough quality filtering comprising two separate rounds. The first was applied to each sample separately and used an automatic doublet detection method, and the second was applied to the merged samples at the curated annotation stage and aimed at detecting and eliminating cells expressing inconsistent markers.

The code to reproduce the analysis is stored at <https://github.com/Papatheodorou-Group/HELMSLEY_Crohn_scRNA-Seq> and will be publicly available at the date of publication.

### Single-cell GEMs library generation (10X Chromium)

The single-cell suspension was immediately processed as per 3’ V3.1 chemistries as described below on the 10X Chromium single-cell platform (10X Genomics). The GEMs were generated from cells with reverse transcription mix, gel beads, and oil, loaded on a Chromium Next GEM Chip G. After cDNA amplification, libraries were prepared and single-indexed with Single Index Plate T Set A. Libraries were sequenced on either a NextSeq or NovaSeq Ilumina sequencer, following the manufacturer’s instructions.

### Sample processing

Read alignment and generation of gene-cell count matrices was performed using cellranger count from CellRanger version 7.1.0 on the human reference genome GRCh38 v2024-A. The parameter -expect-cells was set to 10,000 for all samples.

# Per-sample quality filtering

In the first filtering step, samples were analysed separately using Seurat version 4.0.5, as described below. Here, per-sample clustering was only performed with the aim of detecting low-quality cells, which should be removed prior to doublet detection with DoubletFinder, as per the authors’ recommendations. UMI counts were normalised using the formula $\frac{1e5(x+1)}{n}$ and then log-transformed (natural logarithm). Dimensional reduction was done on the top 5,000 highly variable genes detected using the vst method. A cell-cycle score was computed using the Seurat function CellCycleScoring with default parameters and regressed out. Principal component analysis (PCA) was performed on the z-scores of the log-normalised counts of highly variable genes. A total of 50 principal components (PCs) were computed. The optimal number *n* of PCs to retain for clustering was defined as the minimum *n* such that the standard deviation explained by the *n*th PC exceeds 50% of the average across the 40th to 50th PCs. A first round of clustering was performed with the Louvain method on the selected PCs with a number of neighbours in the k-nn graph *k*= 30, by varying the resolution parameter from 0.5 to 1.2 in steps of 0.1. Differential expression was run between each cluster and its complement with FindMarkers using the MAST method with default parameters. To remove low-quality cells, we looked at mitochondrial gene expression. Instead of setting an arbitrary cut-off on the fraction of mitochondrial transcripts, we instead removed clusters showing upregulation of mitochondrial genes exclusively. Using this approach, we avoided choosing an arbitrary value for mitochondrial transcript fraction and instead used all (variable) gene expression information to identify similarly low-quality cells that should be discarded altogether, which resulted in a cleaner result for downstream analysis. Highly variable gene selection, PCA, and PC selection were re-computed using the same strategy as above, but on the filtered samples, so that gene expression variation was not influenced by low-quality cells’ transcriptomes. Doublets were predicted using DoubletFinder version 2.0.3 on the same PC space using a doublet percentage of 7.6% for all samples considered.

### Automatic cell labelling

To build a ‘reference’ dataset for automatic cell annotation, we leveraged the Gut Cell Atlas published by Elmentaite *et al* (2021) [37]:

(<https://cellgeni.cog.sanger.ac.uk/gutcellatlas/Full_obj_raw_counts_nosoupx_v2.h5ad>). We selected adult and paediatric samples, which comprise 153,961 cells. Then we computed a latent representation of the reference gene expression space with scvi-tools (1.3.3) using scVI with parameters hvg_method=‘seurat3’, ngenes=5000 and batch_key=‘Diagnosis’. Semi-supervised embeddings were obtained from the reference scVI model with scANVI, using either the broad (‘category’) or the fine-grained (‘Integrated_05’) cell type annotation from GCA. Reference models were trained for 20 epochs and with 100 samples per label. We defined as ‘query’ the union of the terminal ileum scRNA-Seq samples after quality filtering, trained the query model on the reference model with weight_decay=0.0 for 200 epochs, and predicted cell-type labels using the reference annotation. Here, a label ‘score’ for a cell was a value ranging from 0 to 1 and was computed for each cell type; the inferred annotation corresponded to the cell type with the highest score.

### Curated cell annotation

Terminal ileum (query) samples for the same condition were merged, and clusters were computed using the procedure described in ‘Sample processing and quality filtering’. Using the automatic cell labelling and doublet detection procedure, we obtained a predicted cell type label and doublet fraction for each cluster. Clusters were either merged or subclustered when the assignment to a cell type was dubious. A second cell filtering step was performed by evaluating doublet fraction information and marker gene expression (i.e. joint expression of markers of different cell types). We believed that cells predicted as doublets would show an intermediate transcriptional profile between two distinct cells, and therefore cells transitioning from one state to another may be incorrectly labelled as doublets. In contrast, because we labelled a fixed fraction of cells as doublets, cells showing a transcriptional profile very similar to that of predicted doublets but that could have exceeded the number of expected doublets thus might incorrectly be labelled as singlets. For these reasons, we decided to use three pieces of information: the doublet label as predicted by DoubletDetection, the cluster assignment, and the automatic cell-type label. Clustering is useful for detecting doublets that are not captured by the algorithm but still show very high transcriptome similarity to doublet-labelled cells and, as such, are clustered together; second, cells expressing inconsistent cell-type markers can be captured within clusters composed of cells that are assigned a mixture of cell-type labels. By performing doublet filtering on the merged samples, we aimed to increase the power of the analysis. We applied a two-step procedure to filter out doublets: (i) remove an entire cluster if its doublet fraction exceeds 70% and (ii) if the total number of expected doublets *N* is higher than the number of cells already removed *n*, then remove the *N-n* cells with a top doublet score. We did not filter out doublets at this stage for CD samples, as a gene expression pattern corresponding to a transitional cellular state could be incorrectly assigned a high doublet score. Then we refined the cell-type annotation from the automatic labelling. When the annotation into a fine-grained cell type was unclear, a subcluster was either (i) filtered out, if it expressed inconsistent markers (and thus considered as doublet-containing), or (ii) assigned a higher-level annotation label. We obtained a total of 25,472 cells for the four normal/control samples and 32,083 cells for the three CD FSL samples, which were annotated into eight cell-type categories and 29 fine-grained cell types.

**Compositional modelling**

The cell-type compositions of CD and normal control samples were modelled using a Bayesian multinomial framework implemented in scCODA (0.1.9) [38]. The model was fit using No-U-Turn Sampling (NUTS), with a target acceptance probability of 0.9 for heuristic step size adaptation and a maximum tree depth of 12, otherwise using default hyperparameters. Sampling was run for 4,000 warm-up iterations and 7,000 sampling iterations. MAST cells were specified as the baseline category to enforce model identifiability. Final model coefficients (β̃) and inclusion probabilities were then estimated for each non-baseline cell type.

Using the model, posterior samples were drawn for each cell type under both conditions, and a SoftMax transformation was applied to transform drawn values from logit space to compositional probability space. The conditions were contrasted as log_2_[(p*_Crohn’s_* + ε)/(p*_Normal_* + ε)], where ε is a small constant to prevent division by zero. Posterior means and 95% credible intervals of the log₂-transformed values were computed, and a cell type was considered compositionally altered if its credible interval did not include zero.

### Cell-cell interaction analysis

We computed differential receptor–ligand interaction signals between each cell-type pair, comparing normal/control and CD FSL cells, using CellChat 2.1.2 for both the curated cell types and cell-type categories. We used protein–protein interactions from the ‘CellChatDB.human’ database, which contained 3,234 interactions at the time of the analysis.

Cell–cell interactions were identified for each condition separately, as follows. Over-expressed interactions were identified on cell-type pairs where both genes are classified as over-expressed (Wilcoxon rank-sum test), using CellChat functions with default parameters. We computed cell–cell interaction probabilities with the computeCommunProb function, which employs a law of mass function approach to propagate gene expression signals in the protein interaction network, using ‘triMean’ to calculate the average gene expression per cell type. Ligand–receptor probabilities were then summarised at the pathway level using the computeCommunProbPathway function with default parameters. Subsequently, we compared the interactions found between conditions using compareInteractions and netAnalysis_signalingRole_network to quantify differences in signalling strength. Differentially enriched ligand–receptor pairs and pathways were then detected with identifyOverExpressedInteractions and netAnalysis_diffSignaling to identify condition-specific communication behaviour between cell types. Higher-order signalling patterns were identified with identifyCommunicationPatterns, with selectK used to determine the optimum number of patterns determined from silhouette and cophenetic correlation metrics based on the elbow heuristic.

## Comparative pathology workbench co-analysis

For final accurate identification of specific features present in the whole-slide imaged sections as well as confirming the correct annotation status by a pathologist, the images and their annotations were uploaded to an OMERO image server. The uploaded images were then organised and shared using the CPW software (<https://workbench-czi-cpw.mvm.ed.ac.uk/home/>). CPW allowed for creating a grid of all of the images of interest, commenting and generating discussion to ensure accurate representation of the pathological state in CD FSL.

## Statistical analyses

All data were analysed with the Shapiro–Wilk test, in addition Q–Q plots and density plots were generated to determine the data normality. For non-normally distributed data, the Wilcoxon rank-sum and signed-rank tests were used to determine significant differences between the data from normal control versus CD FSL for each ileal wall layer. For multiple comparison analysis, the Kruskal–Wallis test with Dunn’s test of multiple comparisons were used with Bonferroni *post hoc* adjustment.


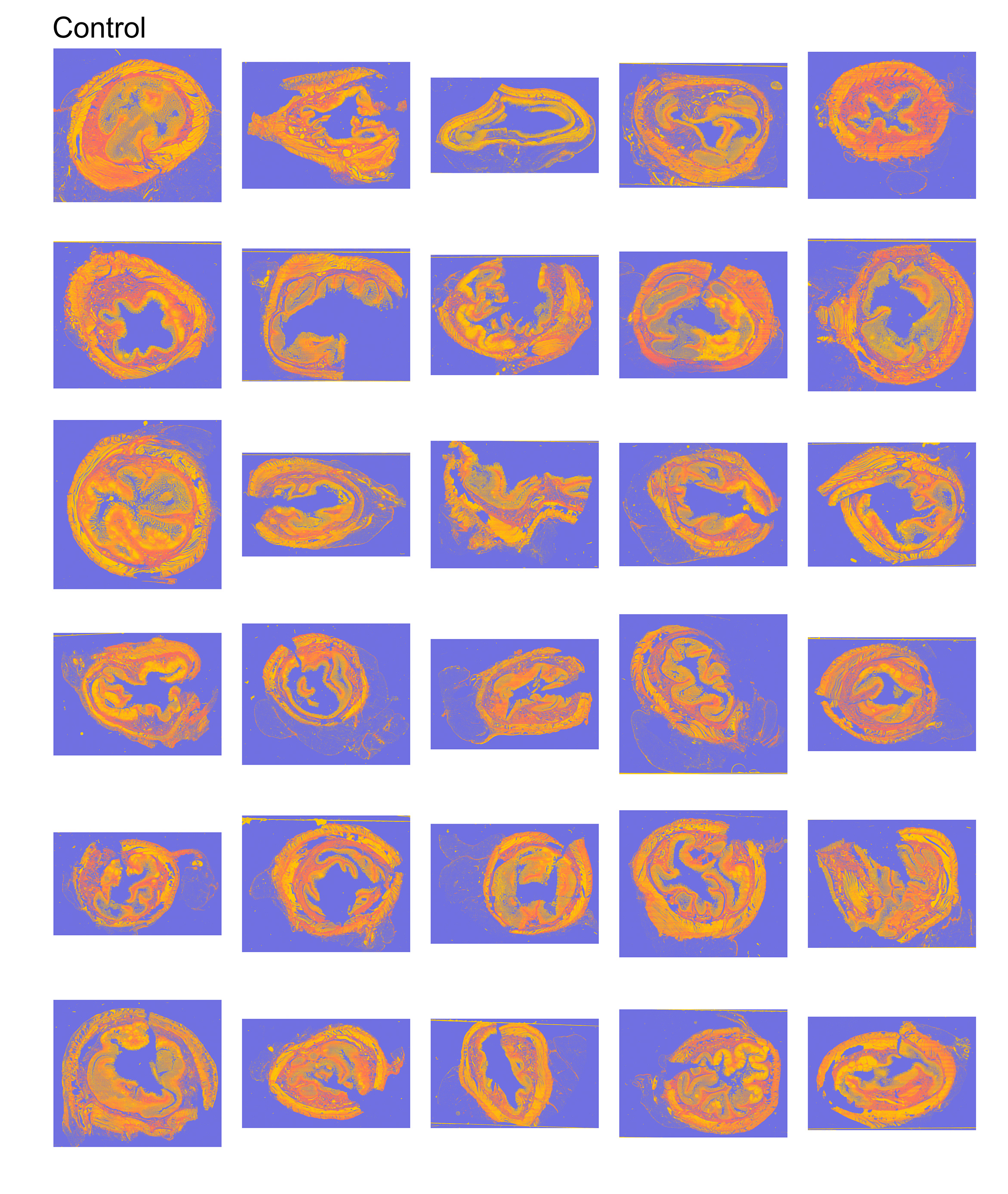


# Figure S1. Exported collagen pixel classifier images for normal control ileal samples. The images show all 30 normal control ileal samples as classified with the collagen pixel classifier. Red, collagen; blue, whitespace (fat cells, empty space); yellow, other (muscle tissue, vasculature and red blood cells, immune cells).


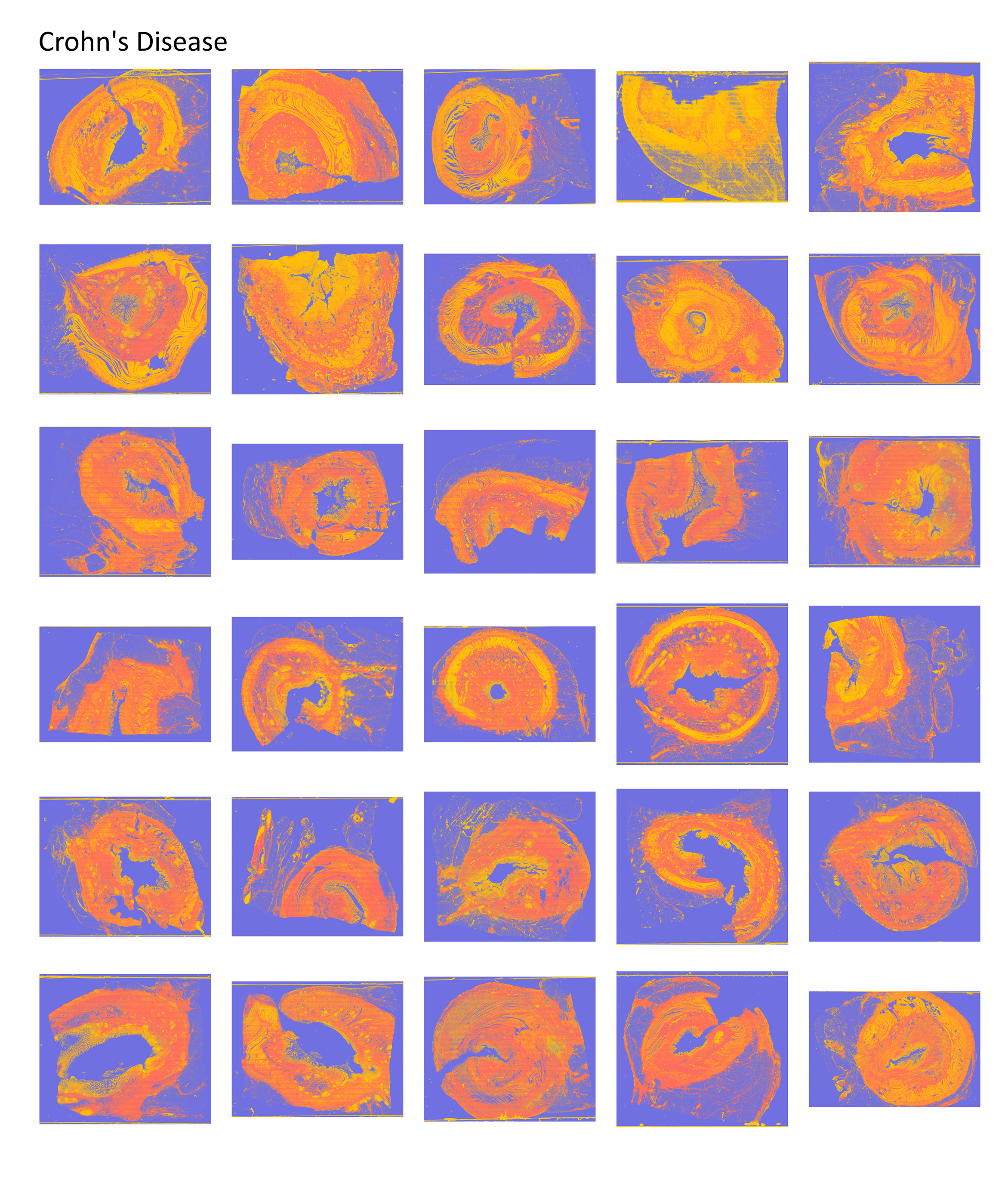


# Figure S2. Exported collagen pixel classifier images for CD ileal FSL samples. The images show all 30 CD ileal FSL samples as classified with the collagen pixel classifier. Red, collagen; blue, whitespace (fat cells, empty space); yellow, other (muscle tissue, vasculature and red blood cells, immune cells).


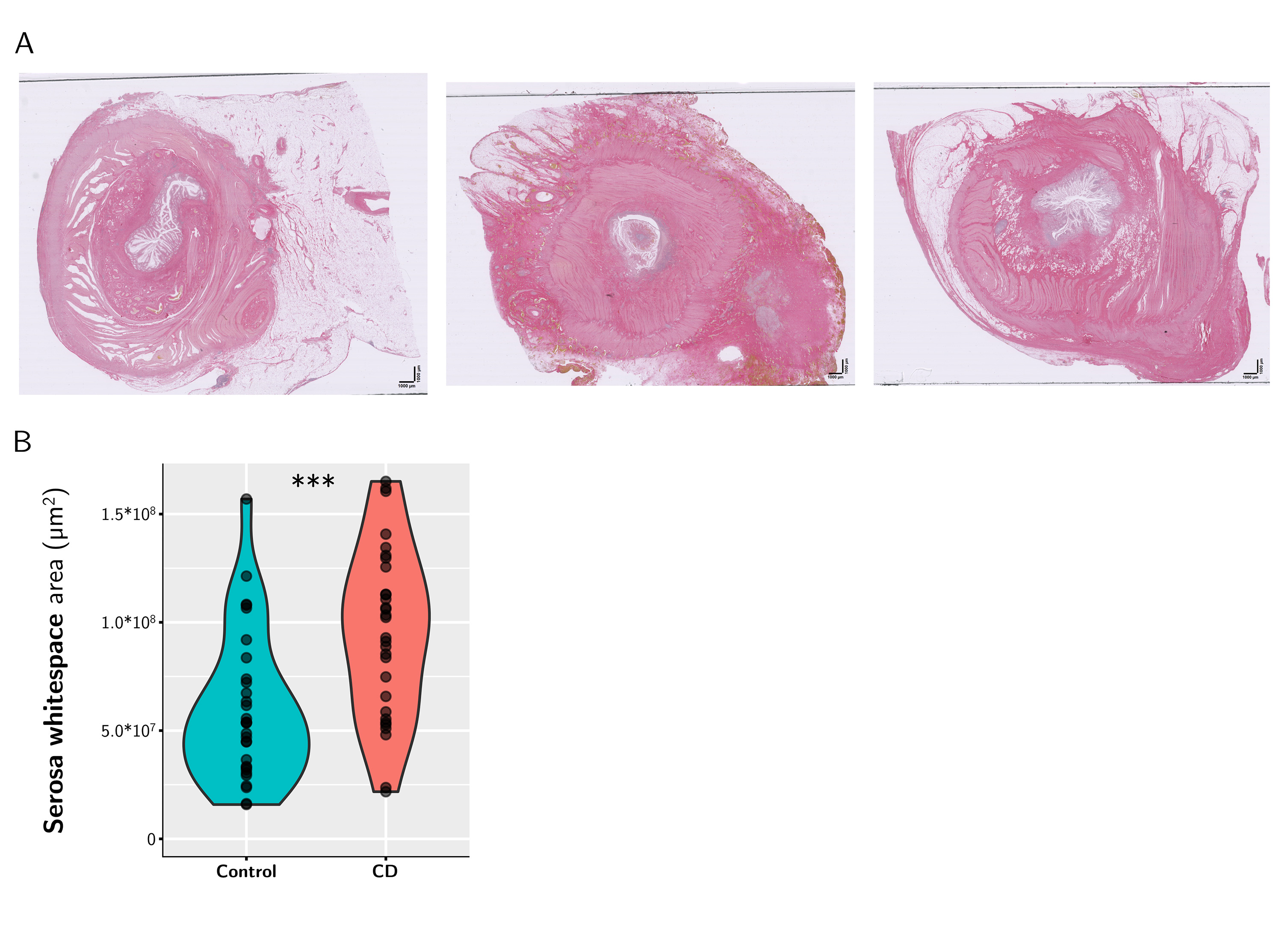


# Figure S3. Fat wrapping or creeping fat formation in serosa of CD FSL samples. (A) Example images of fat wrapping or creeping fat in serosa around the affected ileum in CD FSL samples. (B) Raw serosal whitespace (fat) quantification from the collagen pixel classifier. Normal control (blue) and CD FSL (red) shown as a violin plot; scale bar, 1,000 µm. Statistical significance from non-parametric Wilcoxon rank-sum and signed-rank test: ns – not significant, *p* > 0.05 ns, **p* ≤ 0.05, ***p* ≤ 0.01, ****p* < 0.001, *****p* < 0.0001.


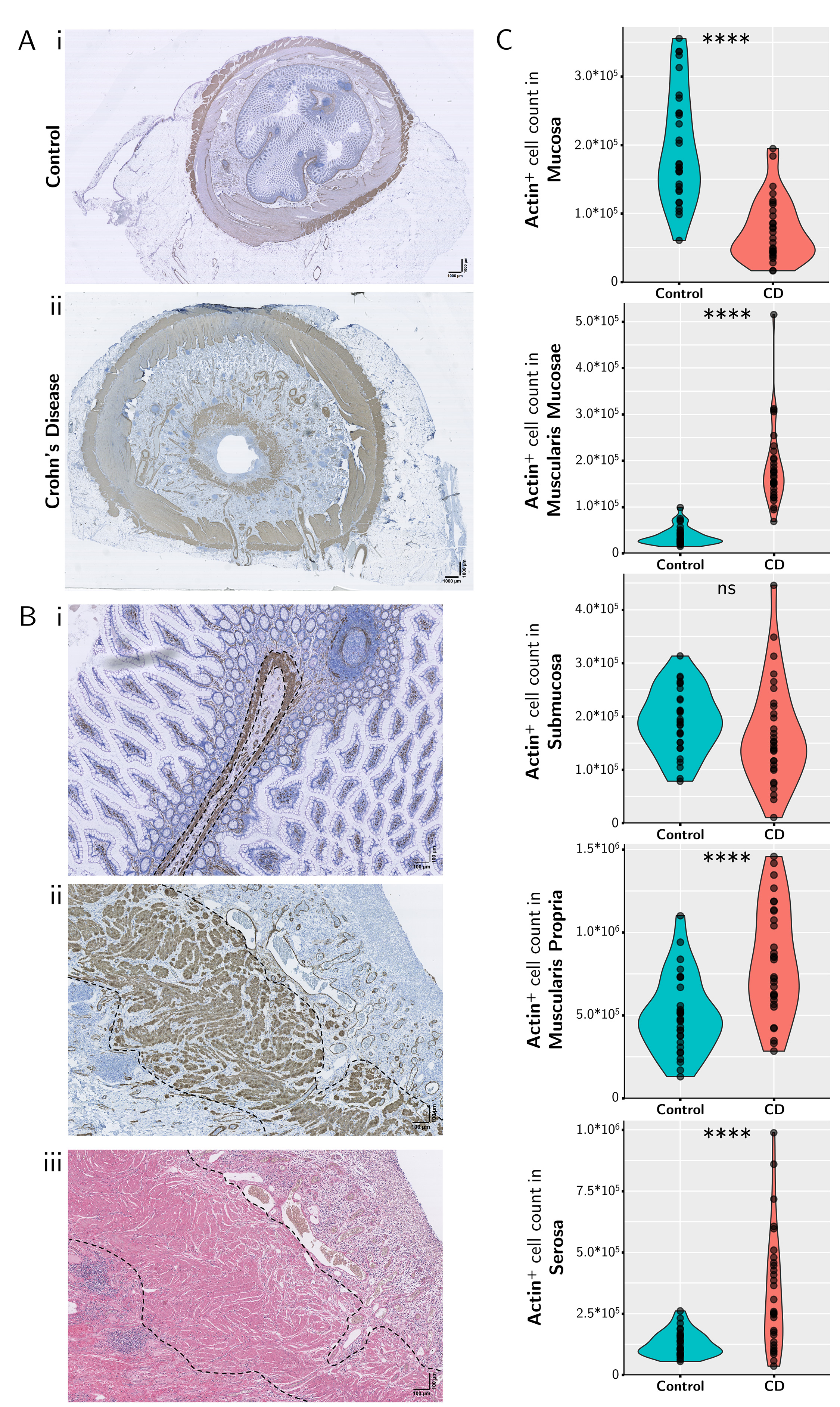


# Figure S4. SMA^+^ IHC staining analysis of smooth muscle cells in normal control and CD FSL samples. (A) Photomicrograph visualisation of IHC stains for SMA^+^ cells in normal control (i) and CD FSL (ii) samples. (B) Photomicrograph visualisation of changes in muscularis mucosae in normal controls (i) compared with CD FSL (muscularis mucosae within black dotted lines) [(ii) (SMA IHC) and (iii) PSR staining of collagen (muscularis mucosae within black dotted lines)]. (C) Quantification of SMA^+^ cell numbers in each ileal layer for normal controls (blue) and CD FSL (red) samples shown as violin plots. Scale bars, 1,000 µm for panel (A) and 100 µm for panel (B). Statistical significance from non-parametric Wilcoxon rank-sum and signed-rank test: *p* > 0.05 ns – not significant, **p* ≤ 0.05, ***p* ≤ 0.01, ****p* < 0.001, *****p* < 0.0001.


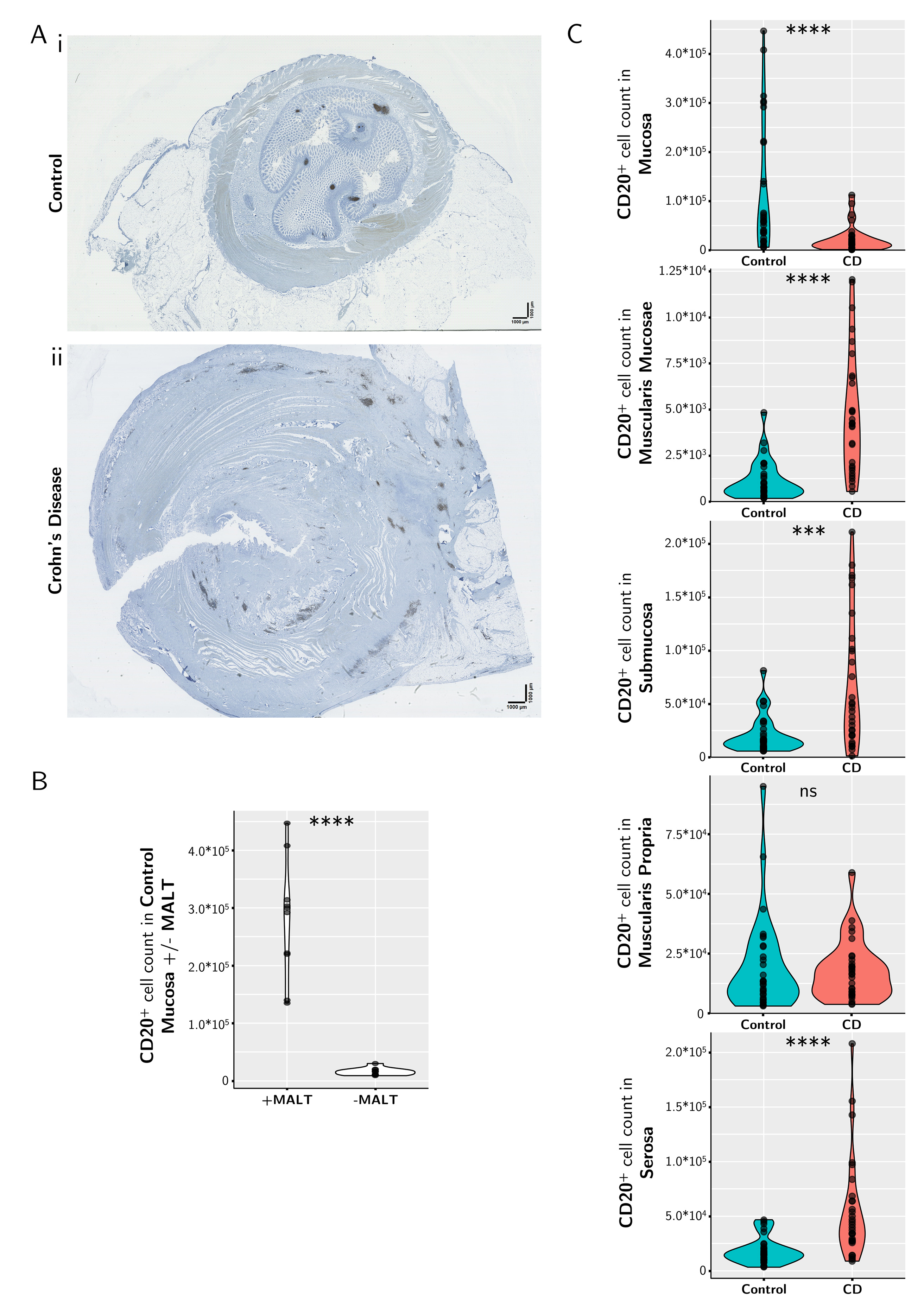


**Figure S5. Quantification and distribution of B lymphocytes.** (A) Photomicrograph visualisation of IHC stains for CD20^+^ B cells in normal control ileum (i) and CD FSL (ii) samples. (B) CD20^+^ B-cell count comparisons between normal control ileum wall mucosa with (+MALT) or without MALT (−MALT). (C) Quantification of CD20^+^ B-cell numbers in each ileal layer for normal control ileum (blue) and CD FSL (red) samples. Scale bar, 1,000 µm. Statistical significance from non-parametric Wilcoxon rank-sum and signed-rank test: *p* > 0.05 ns – not significant, **p* ≤ 0.05, ***p* ≤ 0.01, ****p* < 0.001, *****p* < 0.0001.


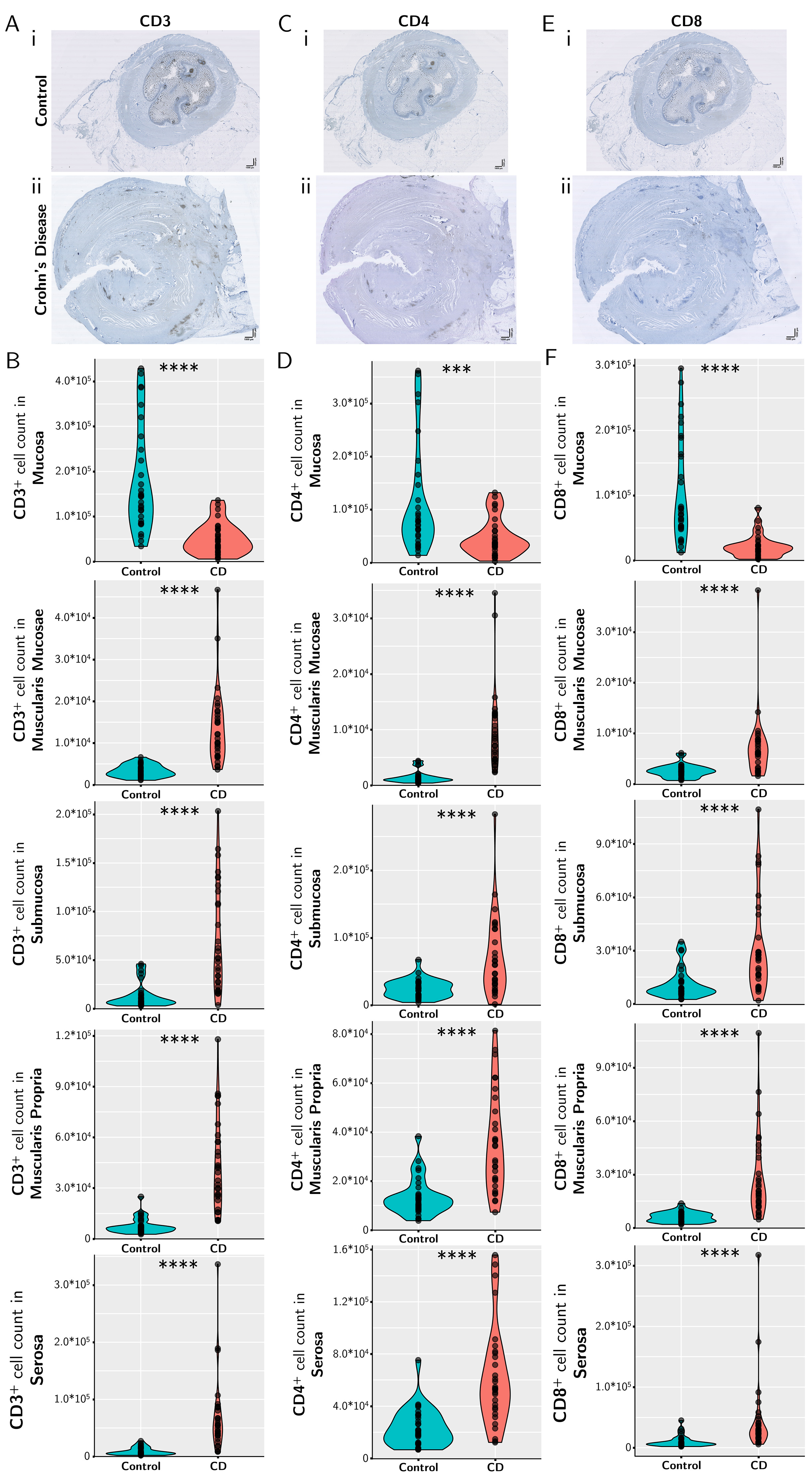


# Figure S6. Quantification and distribution of T lymphocytes. Photomicrograph visualisation of immunohistochemical stains for CD3^+^ (A), CD4^+^ (C), and CD8^+^ (E) T cells in normal control (i) and CD FSL (ii) samples. Quantification of CD3^+^ (B), CD4^+^ (D), and CD8^+^ (F) T-cell numbers in each ileal layer for normal control (blue) and CD FSL (red) samples. Scale bar, 1,000 µm. Statistical significance from non-parametric Wilcoxon rank-sum and signed-rank test: *p* > 0.05 ns – not significant, **p* ≤ 0.05, ***p* ≤ 0.01, ****p* < 0.001, *****p* < 0.0001.


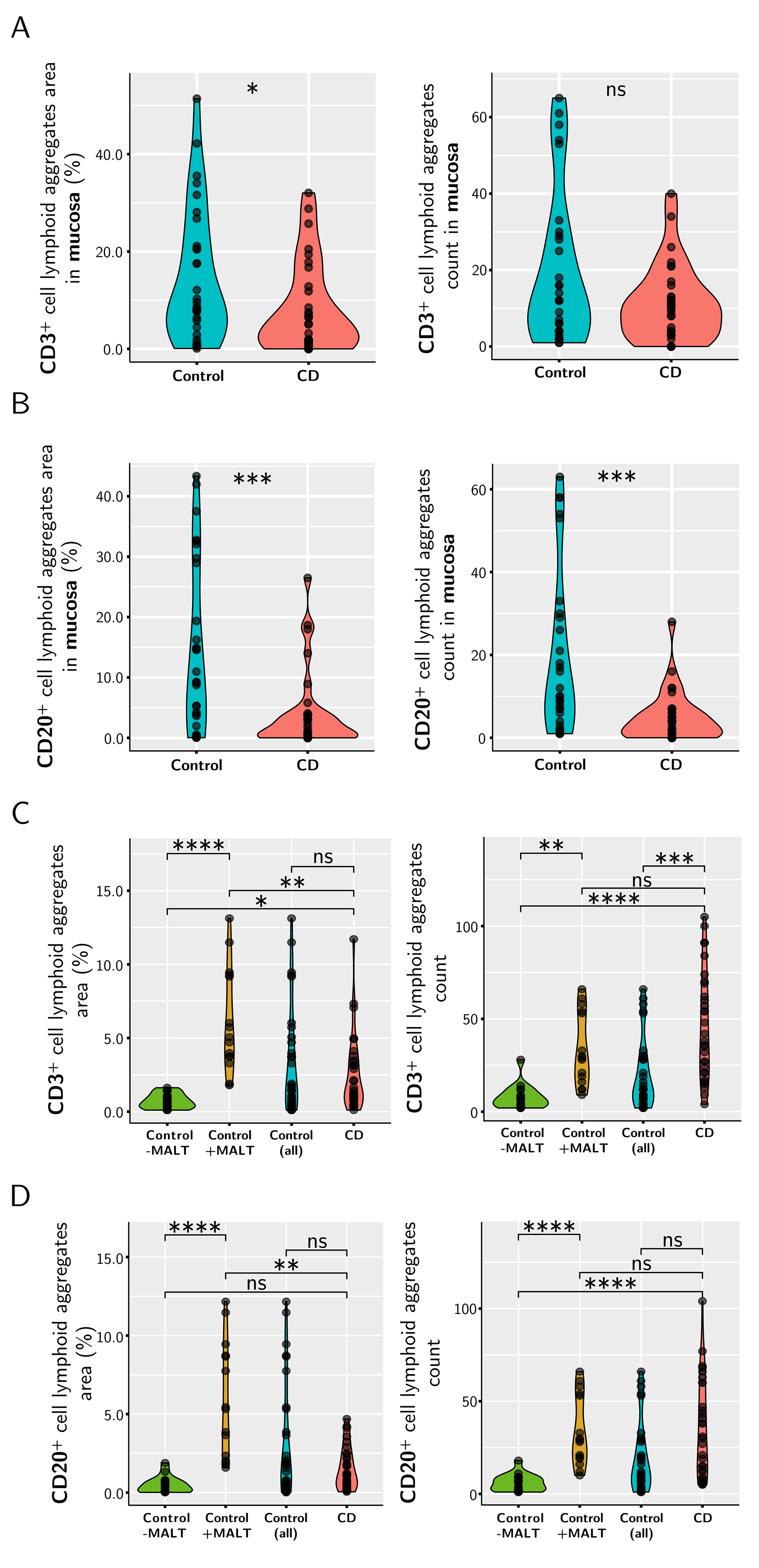


# Figure S7. Lymphoid aggregate quantification in mucosa. Quantification of lymphoid aggregates in mucosa – percentage area (left) and lymphoid aggregate count (right) in (A) CD3+ and (B) CD20+. (C) Comparison of CD3+ lymphoid aggregate percentage area normalised to the layer area (left) and CD3+ lymphoid aggregate counts (right) for normal control ileum (with and without MALT aggregates and combined) compared with CD3+ Crohn’s lymphoid aggregates in CD FSL samples. (D) Comparison of CD20+ lymphoid aggregate percentage area normalised to layer area (left) and CD20+ lymphoid aggregate counts (right) for normal control ileum (with and without MALT aggregates and combined) compared with CD20+ Crohn’s lymphoid aggregates in CD FSL samples. Statistical significance for (A) and (B) from Wilcoxon Rank Sum and Signed Rank Test, and for (C) and (D) from Kruskal–Wallis test with Dunn’s test of multiple comparisons were used with Bonferroni *post* *hoc* adjustment: *p* > 0.05 ns – not significant, **p* ≤ 0.05, ***p* ≤ 0.01, ****p* < 0.001, *****p* < 0.0001.


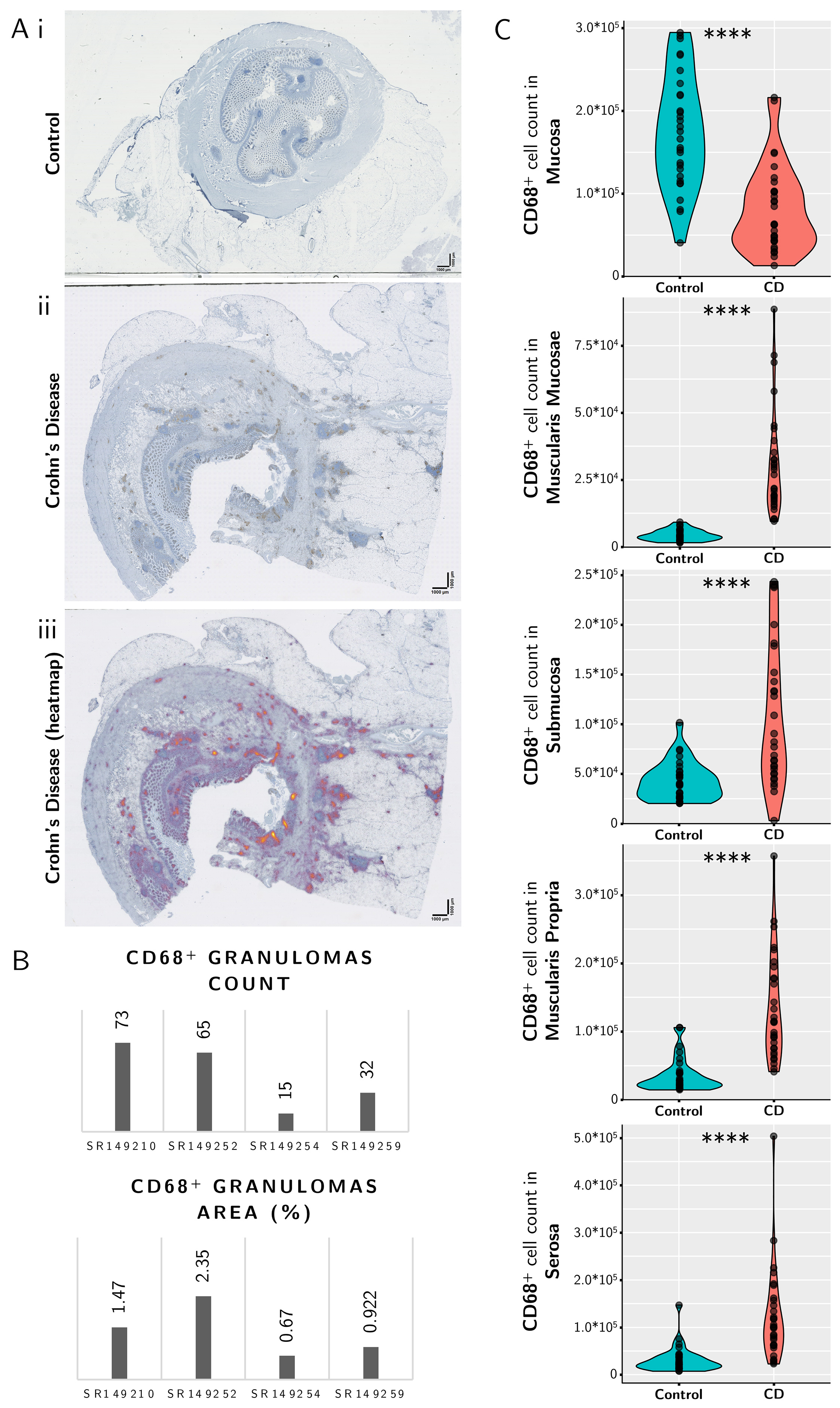


# Figure S8. CD68^+^ macrophage quantification and granuloma identification. (A) Photomicrograph visualisation of immunohistochemical stains for CD68^+^ macrophages in normal control ileum (i) and CD FSL (ii) samples. Granulomas were identified by QuPath density cluster map analysis and found to be present in four samples of CD FSL, visualised using density heatmapping (iii). (B) CD68^+^ granuloma count (upper) and CD68^+^granuloma percentage area (lower) quantification for the four  granuloma-containing CD FSL cases. (C) Quantification of CD68^+^ cell numbers in each ileal layer for normal control ileum (blue) and CD FSL (red) samples. Scale bar, 1,000 µm. Statistical significance from Wilcoxon rank-sum and signed-rank test: *p* > 0.05 ns – not significant, **p* ≤ 0.05, ***p* ≤ 0.01, ****p* < 0.001, *****p* < 0.0001.


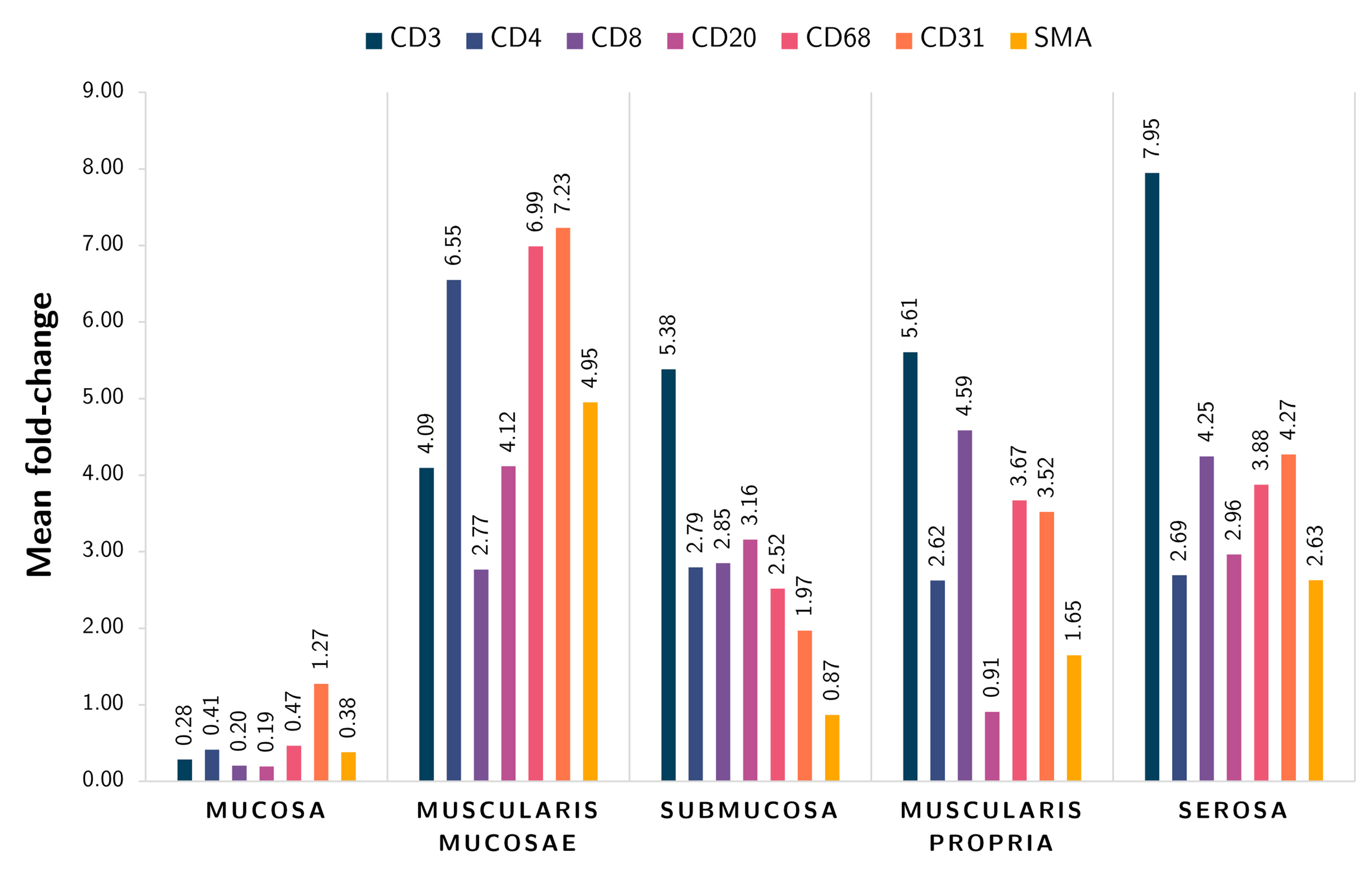


# Figure S9. Bar chart of mean cell number fold-change. Bar chart showing mean fold-changes of values for CD FSL samples over normal control values of different immunohistochemically identified cell types for each intestinal wall layer (CD FSL versus normal control).


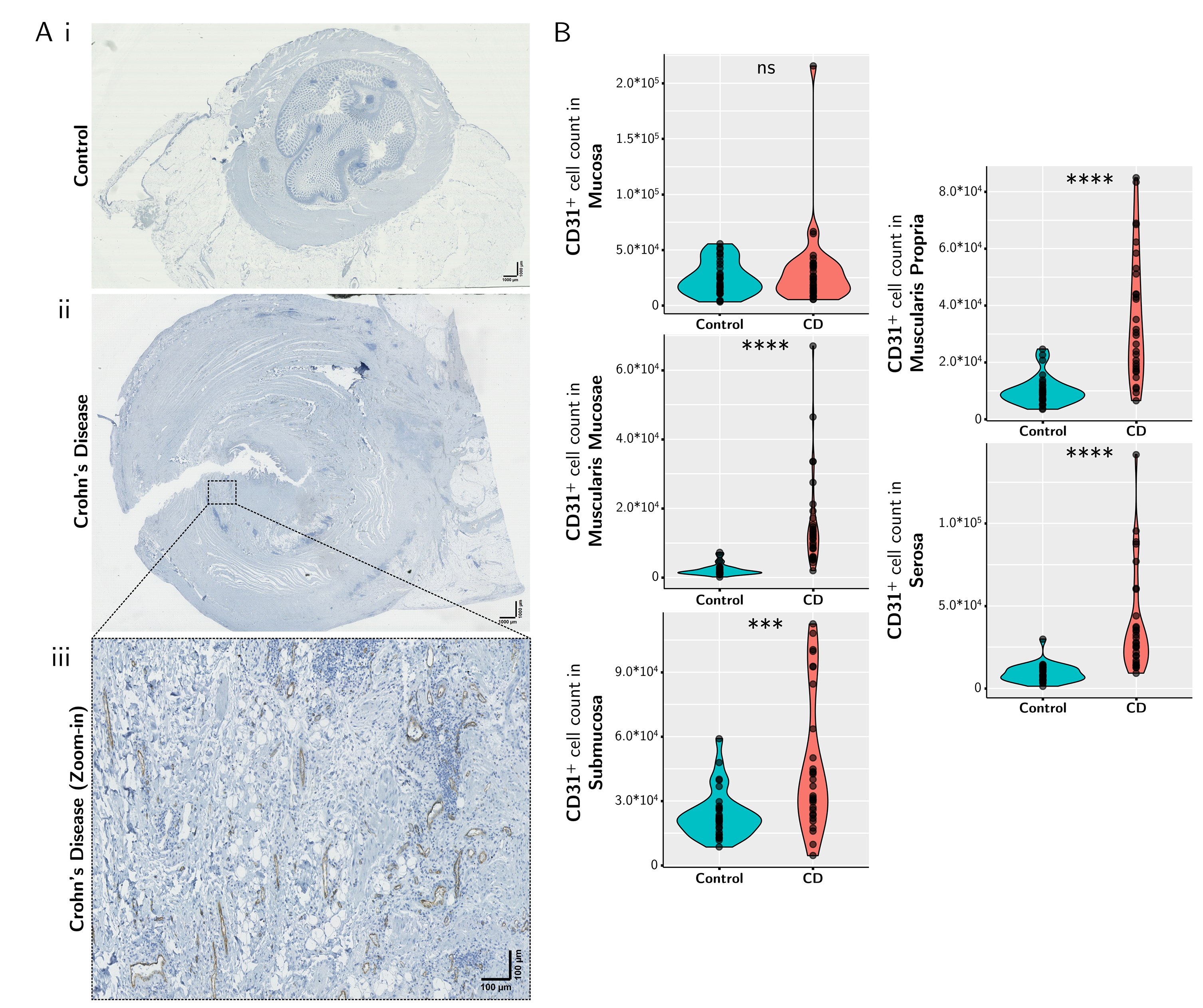


## Figure S10. CD31^+^ endothelial cell population analysis. (A) Photomicrograph visualisation of immunohistochemical stains for CD31^+^ showing endothelial cells mostly forming small blood vessels in normal ileum sample (i) and CD FSLs (ii) with a high-power view of a selected fibrotic area (iii) not containing any Crohn’s lymphoid aggregates in a CD FSL sample. (B) Quantification of CD31^+^ cell numbers in each ileal layer for normal control ileum (blue) and CD FSL (red) samples. Scale bars, (i) and (ii) 1,000 µm and (iii) 100 µm. Statistical significance from Wilcoxon rank-sum and signed-rank test: *p* > 0.05 ns – not significant, **p* ≤ 0.05, ***p* ≤ 0.01, ****p* < 0.001, *****p* < 0.0001.


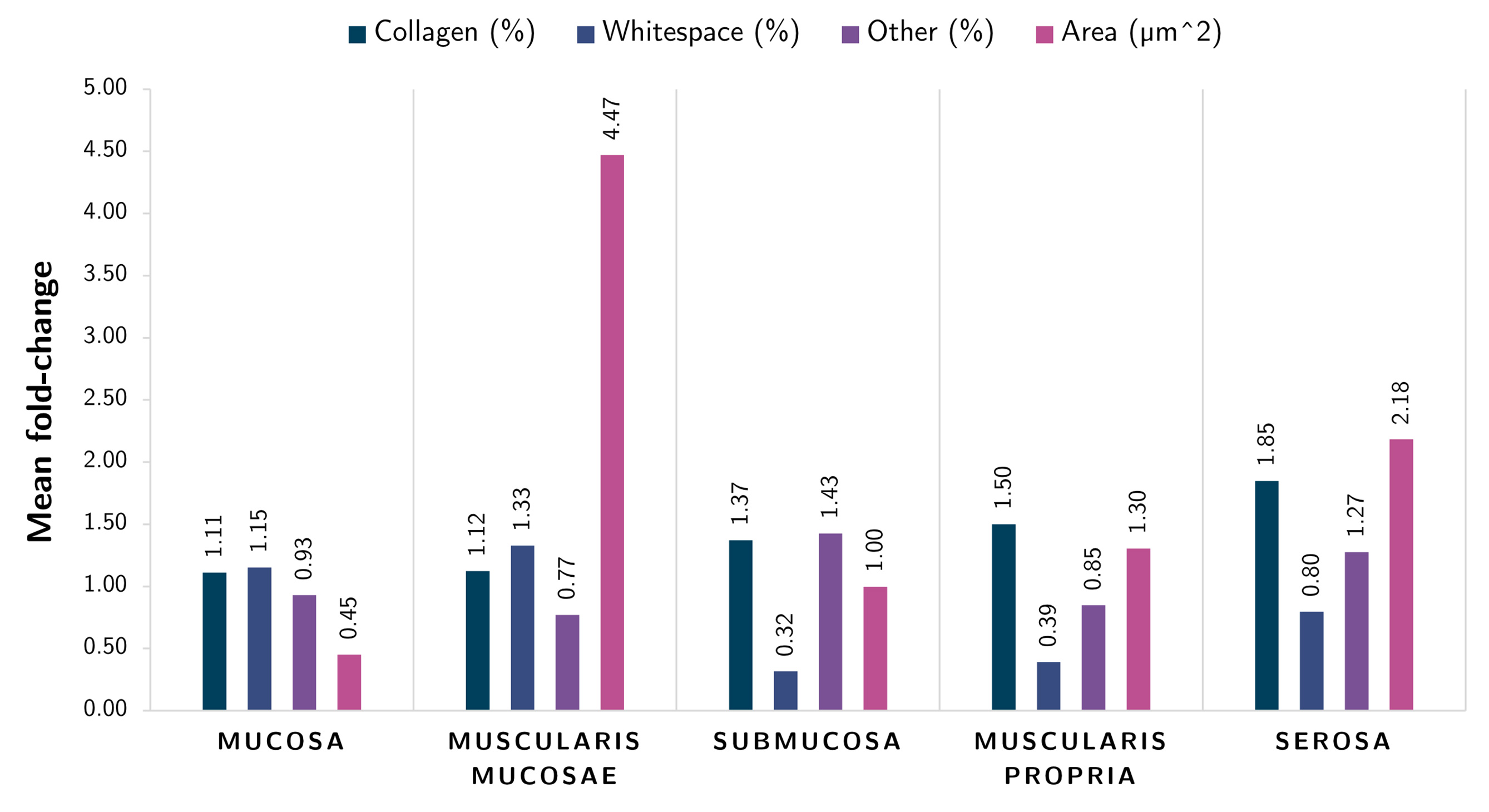


# Figure S11. Bar chart of mean fold-change for collagen (%), whitespace (%), other (%) and layer areas (µm^2^). Bar chart showing mean fold-changes of values for CD FSL samples over normal control values for each parameter (as a percentage) extracted from the pixel classifier for collagen (picrosirius red positive), whitespace (fat and background empty space), other (muscle, vessels, red blood cells, immune cells, other cells), and layer areas (in µm^2^) (CD FSL versus normal control).


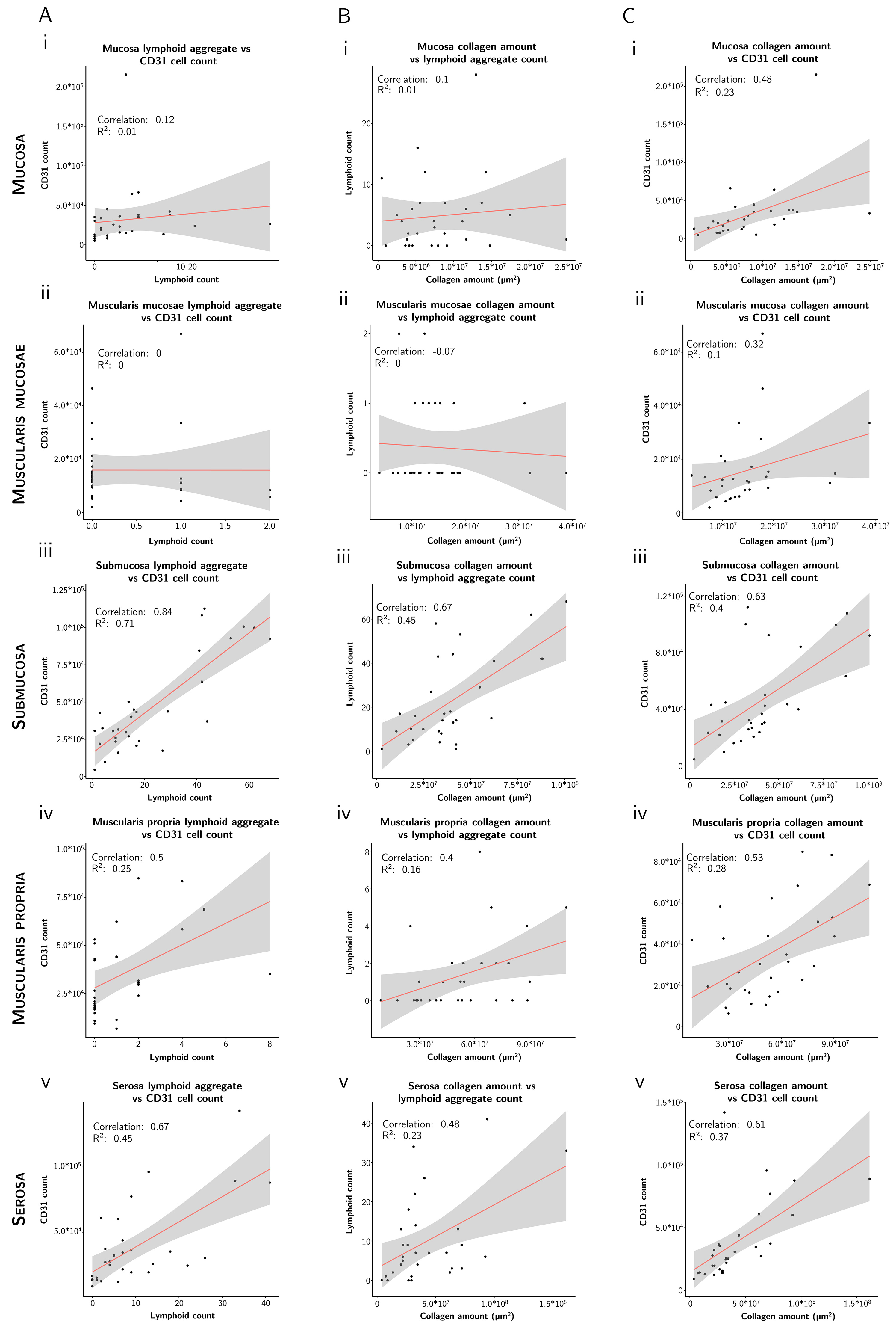


# Figure S12. Correlation analysis between Crohn’s lymphoid aggregate counts, CD31+ cell counts, and amount of collagen (µm^2^). Scatterplots showing correlation analysis of (A) lymphoid aggregate counts versus CD31+ cell counts for all layers, (B) lymphoid aggregate counts versus collagen amounts (as areas in µm^2^) for all layers, and (C) CD31+ cell counts versus collagen amounts (as areas in µm^2^), for all layers, in (i) mucosa, (ii) muscularis mucosae, (iii) submucosa, (iv) muscularis propria, and (v) serosa layers for CD FSL samples. Correlation coefficient (R) and coefficient of determination (*R*^2^) for each plot are included in the top left-hand corners.


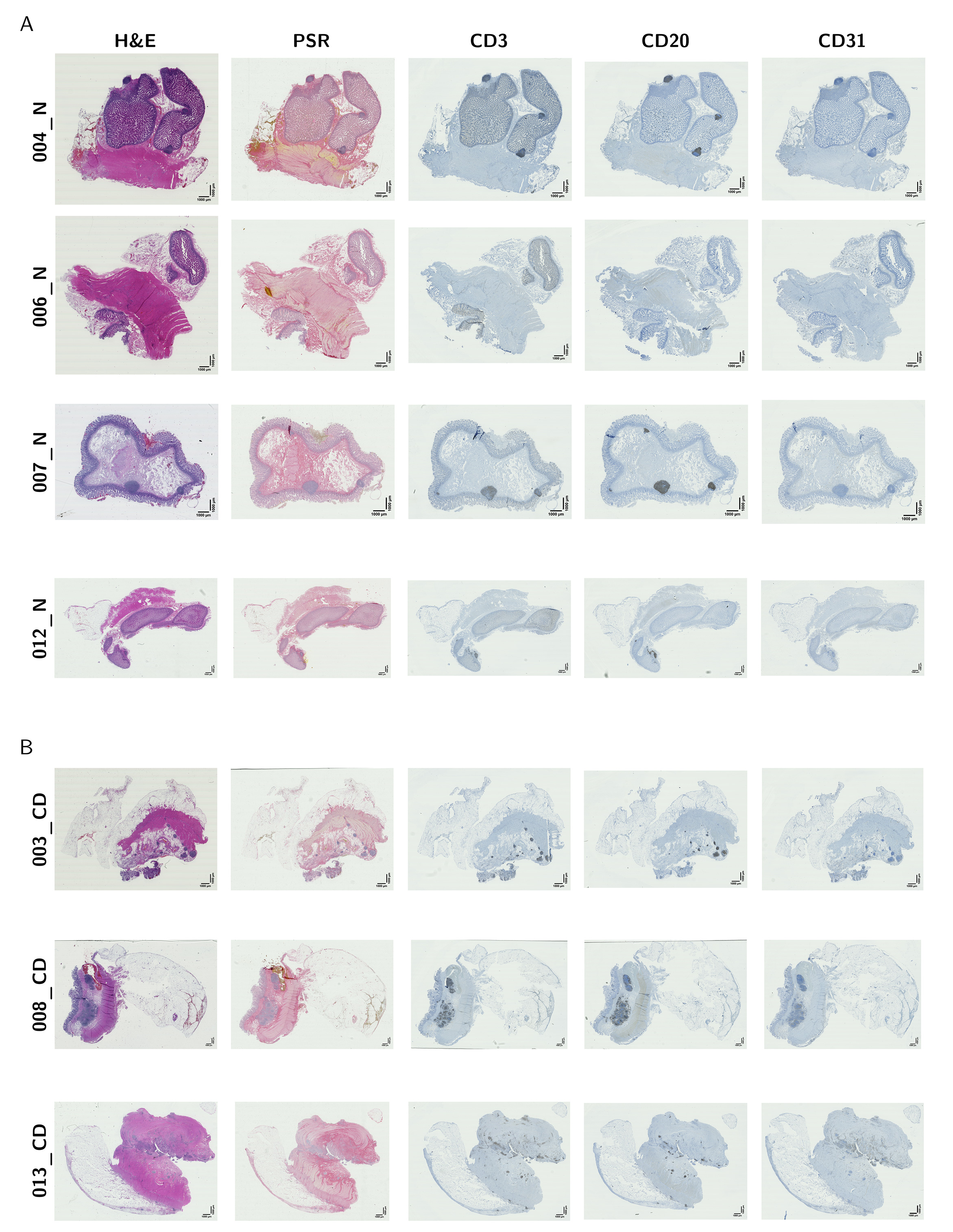
**Figure S13. H&E, PSR, and immunohistochemically stained photomicrograph images of four freshly collected normal control ileum and three freshly collected CD FSL samples used in scRNA-seq analysis.** Images of HE), PSR, and CD3 IHC, CD20 IHC, and CD31 IHC stained sections from (A) normal control ileum and (B) CD FSL ileum samples. Scale bar, 1,000 µm.


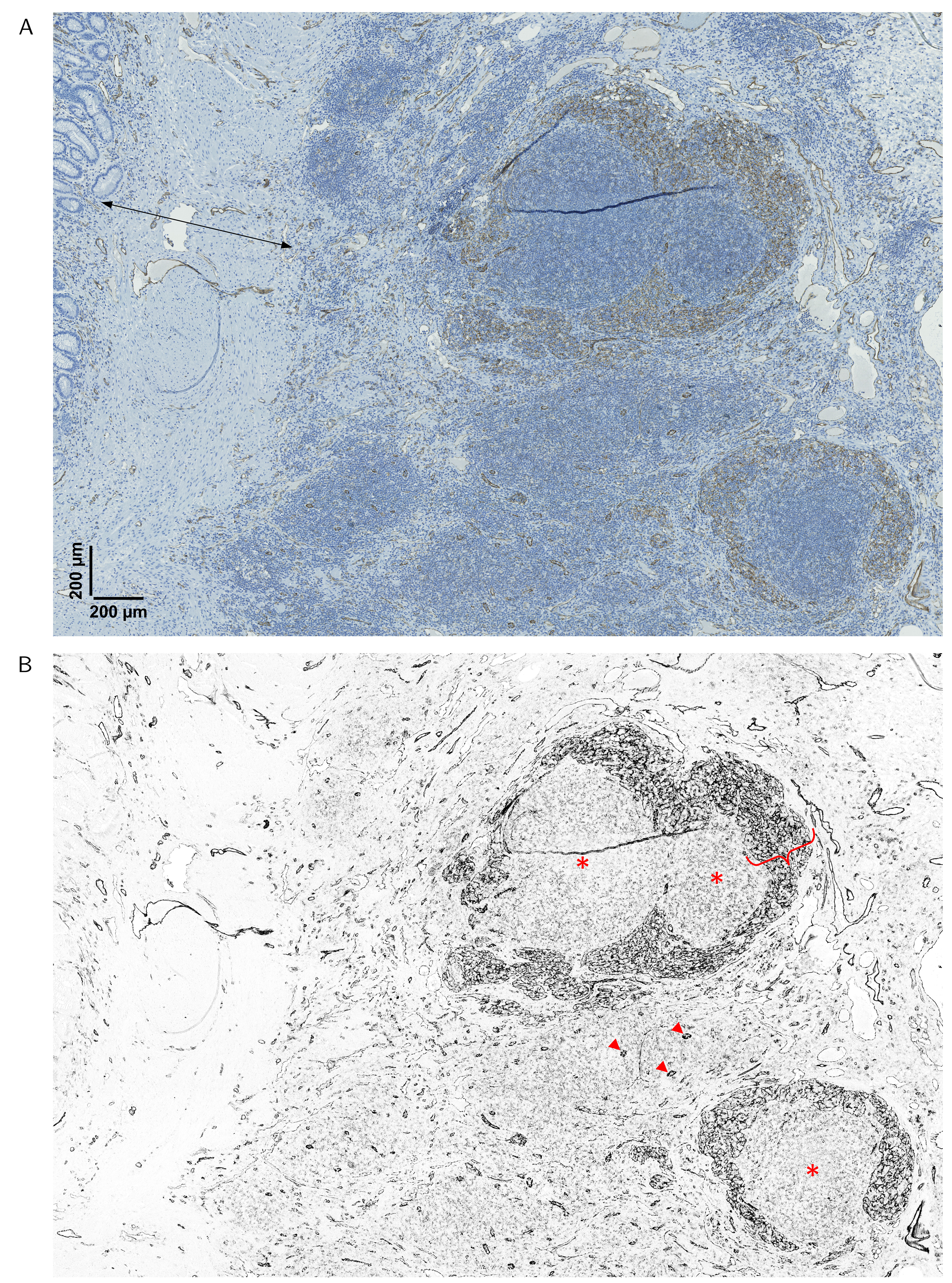


**Figure S14. Photomicrograph images of immunohistochemical stains for CD31+ endothelial cells showing a marked increase and accumulation of CD31+ cells around Crohn’s lymphoid aggregates in freshly collected CD FSL samples used in scRNA-seq analysis.** The photomicrograph images show (A) CD31+ IHC staining with CD31+ endothelial cells stained in brown and (B) QuPath-identified CD31+ cells in black. In the lower panel, red arrowheads indicate small capillary vessels directly adjacent to Crohn’s lymphoid aggregates; curly brackets indicate accumulation of individual and clusters of CD31+ endothelial cells around a Crohn’s lymphoid aggregate; * indicates centre of Crohn’s lymphoid aggregates containing CD3+ T and CD20+ B cells; black double-headed arrow (upper panel) shows expansion of the muscularis mucosae containing CD31+ small vessels. Scale bar, 200 µm.


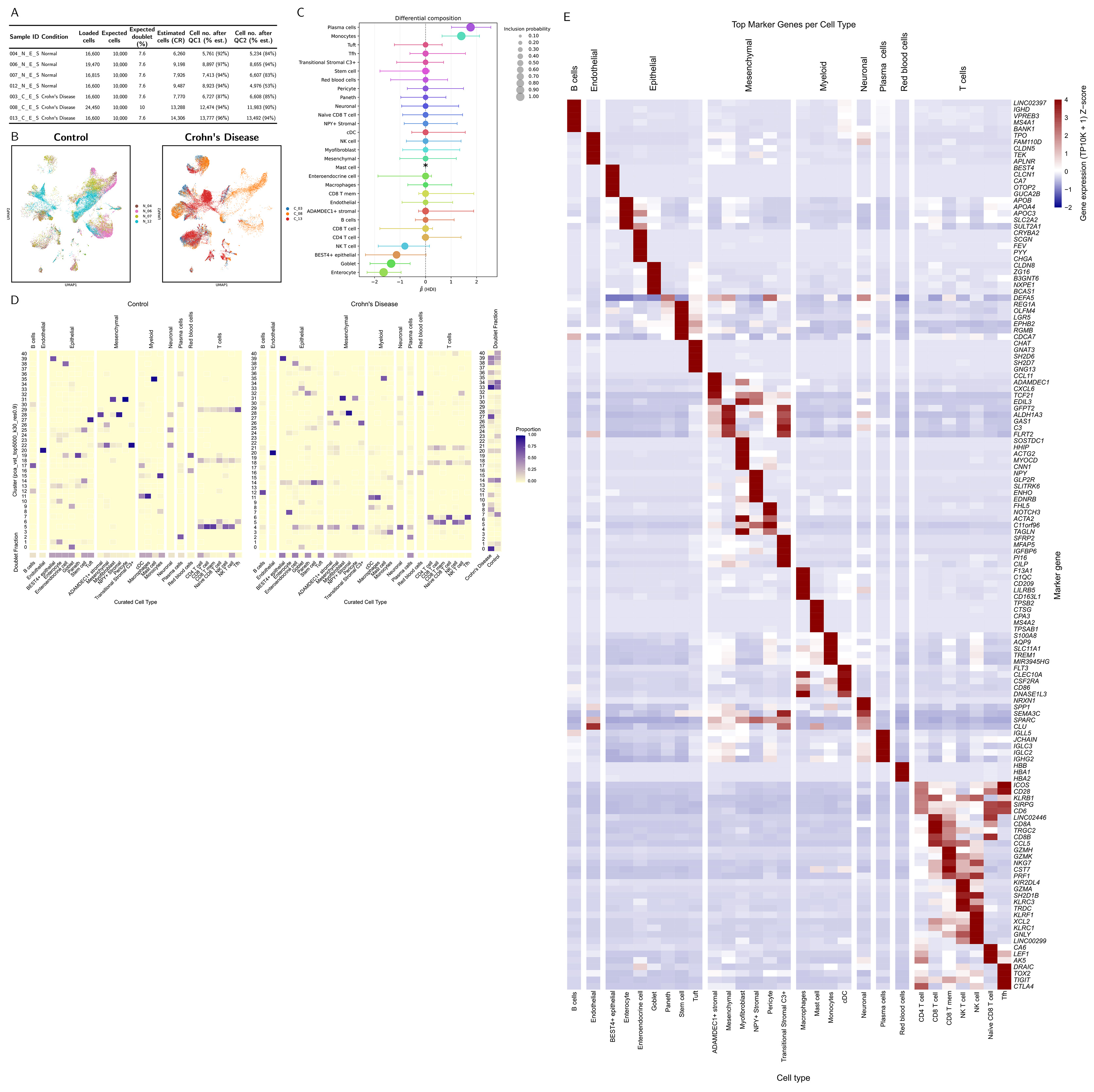


**Figure S15. Overview of scRNA-seq data with table of QC metrics, UMAP visualisations, compositional model coefficients, and marker genes of four normal control and three CD FSL ileum samples.** (A) Table showing QC parameters and number of surviving cells at each stage of QC preprocessing. (B) UMAP embeddings coloured by sample type and identification number for normal control samples (left) and for CD FSL samples (right). (C) Chart showing final model coefficients $\tilde{\boldsymbol{\beta}}$ from differential cell type compositional modelling with scCODA, with mast cells (indicated by *) used as baseline category. Bars represent 95% highest density interval (HDI), and point size reflects the inclusion probability of each coefficient after FDR adjustment (*q* = 0.05). (D) Proportion of coinciding cells between unsupervised clusters and curated cell-type identities and fraction of predicted doublets within each cluster and cell type. (E) Top five most expressed marker genes per curated cell type identified using the Wilcoxon rank-sum test and ranked by Z-normalised gene expression. Colour scale represents Z-score, from below-average (blue) to above-average (red) expression relative to gene-wise mean across all cells.


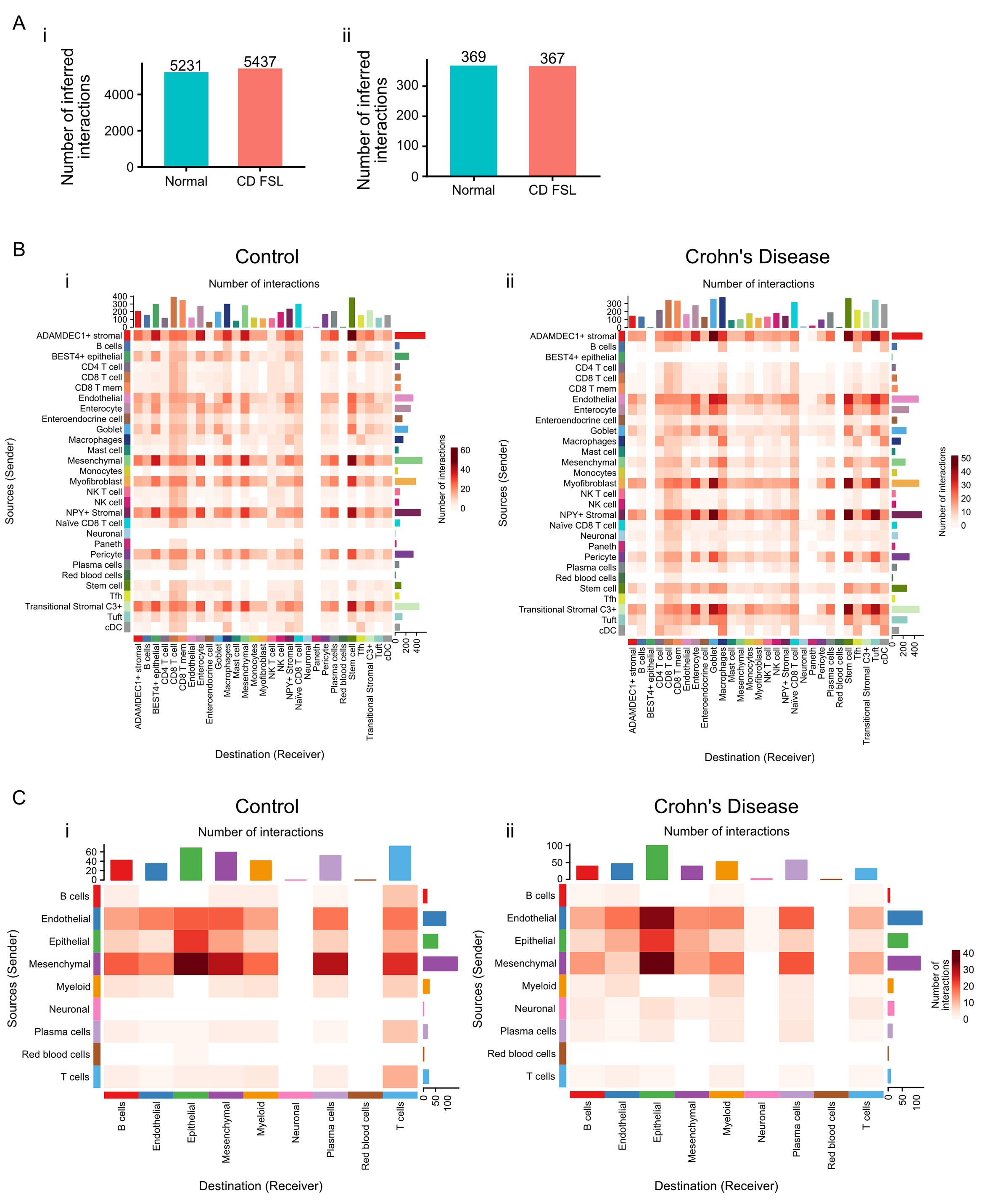


**Figure S16. Analysis of ligand–receptor signalling interactions between pairs of cell types using CellChat.** (A) Bar graphs showing total number of inferred interactions for normal control and CD FSL samples for (i) all of the individual curated cell types and (ii) the lower-granularity cell-type categories. (B) Heatmaps showing numbers of interactions between each of the individual curated cell types for (i) normal control samples and (ii) CD FSL samples, with source/sender signalling cells on the *y*-axis and destination/receiver cells on the *x*-axis. (C) Heatmaps showing numbers of interactions between higher-order cell categories for (i) normal control samples and (ii) CD FSL samples, with source/sender signalling cell categories on the *y*-axis and destination/receiver cell categories on the *x*-axis.


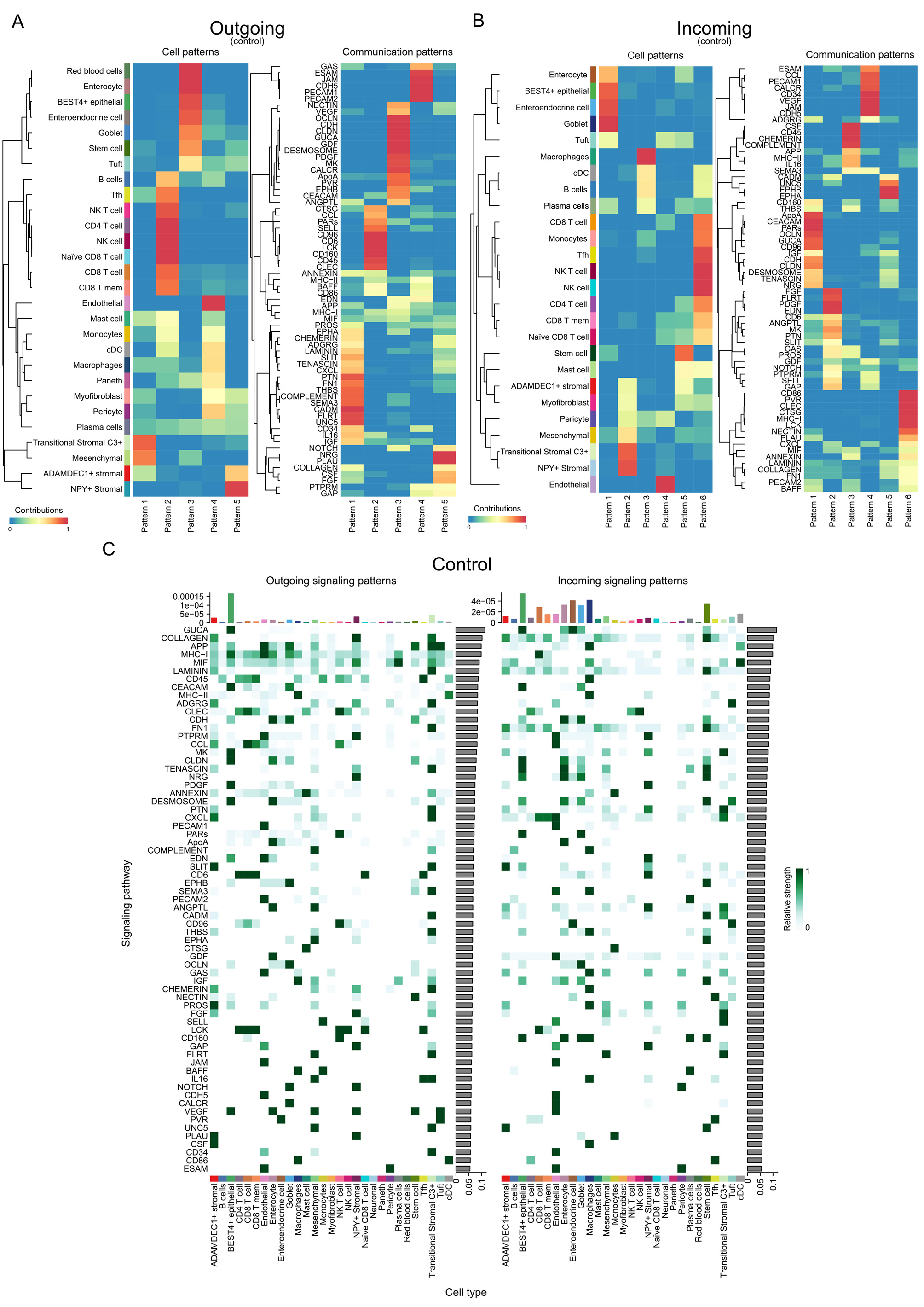


## Figure S17. Higher-order signalling patterns between cells in normal control sample data. (A and B) Heatmaps showing individual curated cell types or signalling pathways (*y*-axis) and signalling interaction communication patterns (*x*-axis) for (A) outgoing signals, with cell patterns on the left and communication patterns on the right, and (B) incoming signals, with cell patterns on the left and communication patterns on the right, in normal control sample data. (C) Heatmaps showing signalling pattern interactions (*y*-axis) and their strengths, related to the most common signalling pathways present for each curated cell type (*x*-axis), with outgoing signalling patterns on the left and incoming signalling patterns on the right for normal control sample data.


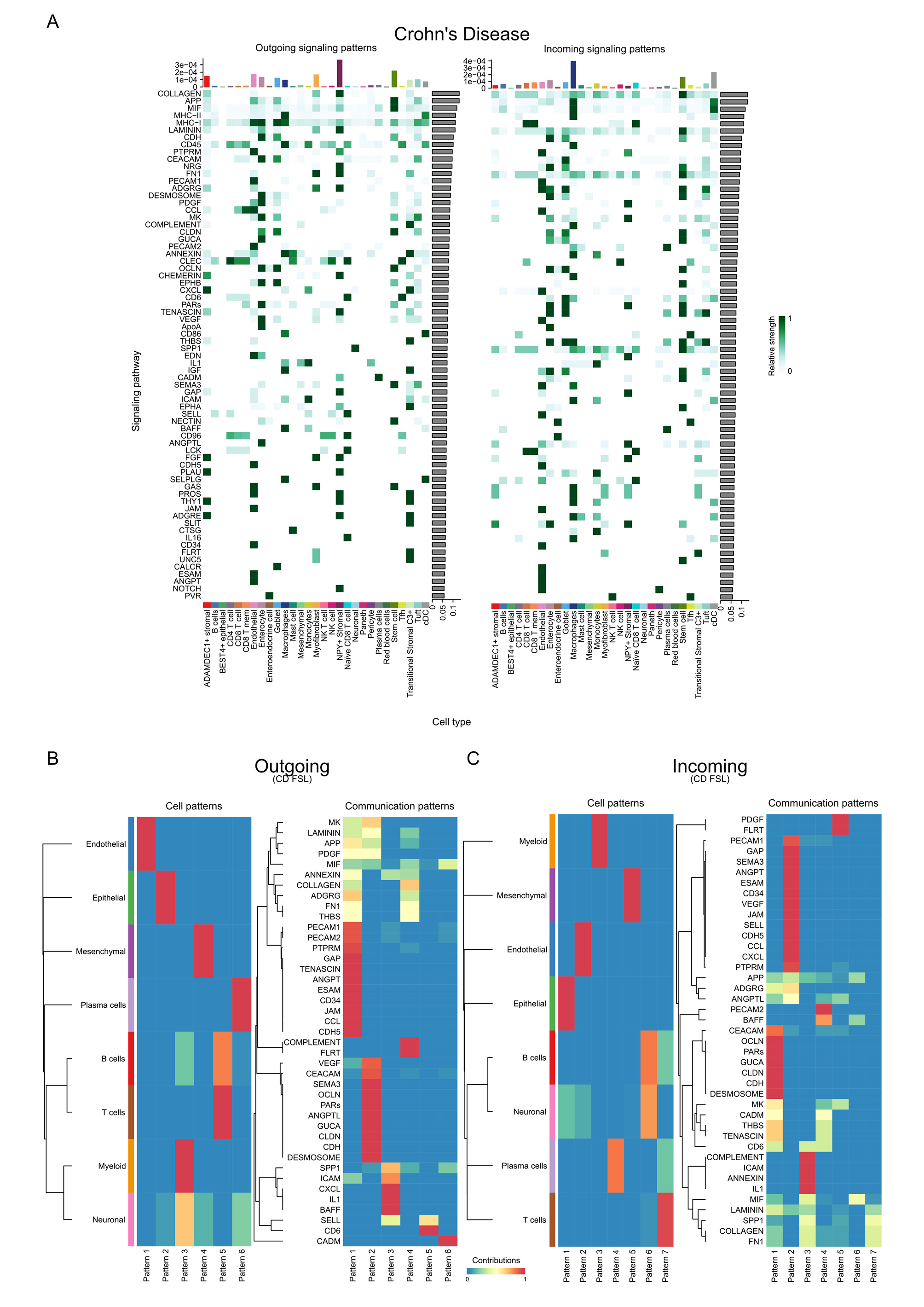


**Figure S18. Higher-order signalling patterns between cells in CD FSL sample data.** (A) Heatmaps showing signalling pattern interactions (*y*-axis) and their strengths, related to the most common signalling pathways present for each curated cell type (*x*-axis), with outgoing signalling patterns on the left and incoming signalling patterns on the right for CD FSL sample data. (B and C) Heatmaps showing higher-order cell categories or signalling pathways (*y*-axis) and signalling interaction communication patterns (*x*-axis) for (B) outgoing signals, with cell patterns on left and communication patterns on right, and (C) incoming signals, with cell patterns on left and communication patterns on right, in CD FSL sample data.


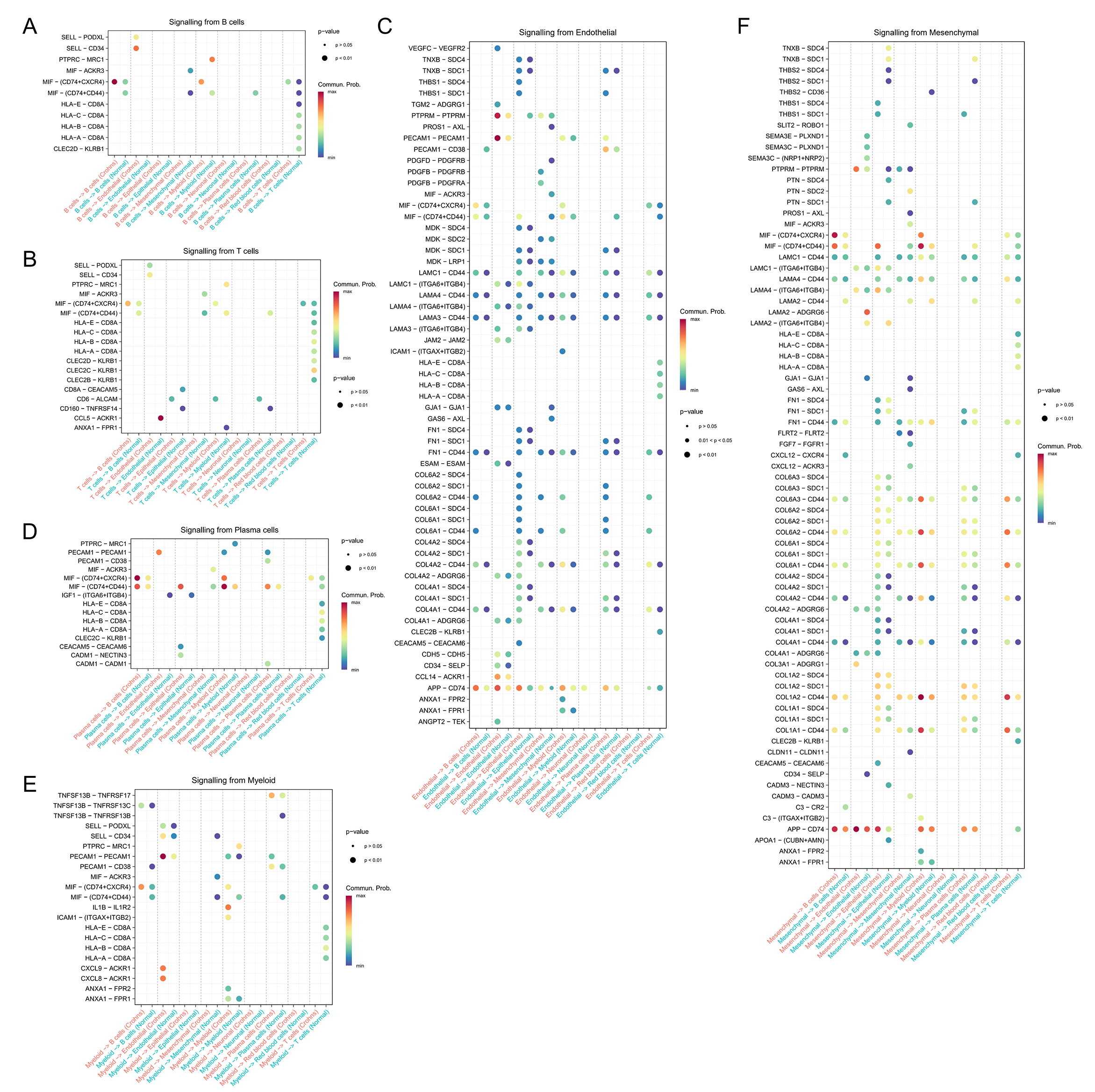


## Figure S19. Ligand–receptor signalling interaction patterns between cell-type categories in normal control and CD FSL sample data. (A–F) Dot plots showing specific ligand–receptor pathway signalling (*y*-axis) between a variety of selected pairs of cell-type categories (*x*-axis, CD FSL in red, normal controls in blue) for signalling from (A) B cells, (B) T cells, (C) endothelial cells, (D) plasma cells, (E) myeloid cells, and (F) mesenchymal cells. Point colour indicates min-max scaled communication probability of that signalling pathway taking place between specific pairs of cell categories (*x*-axis), and point sizes represent *p* value of interaction.


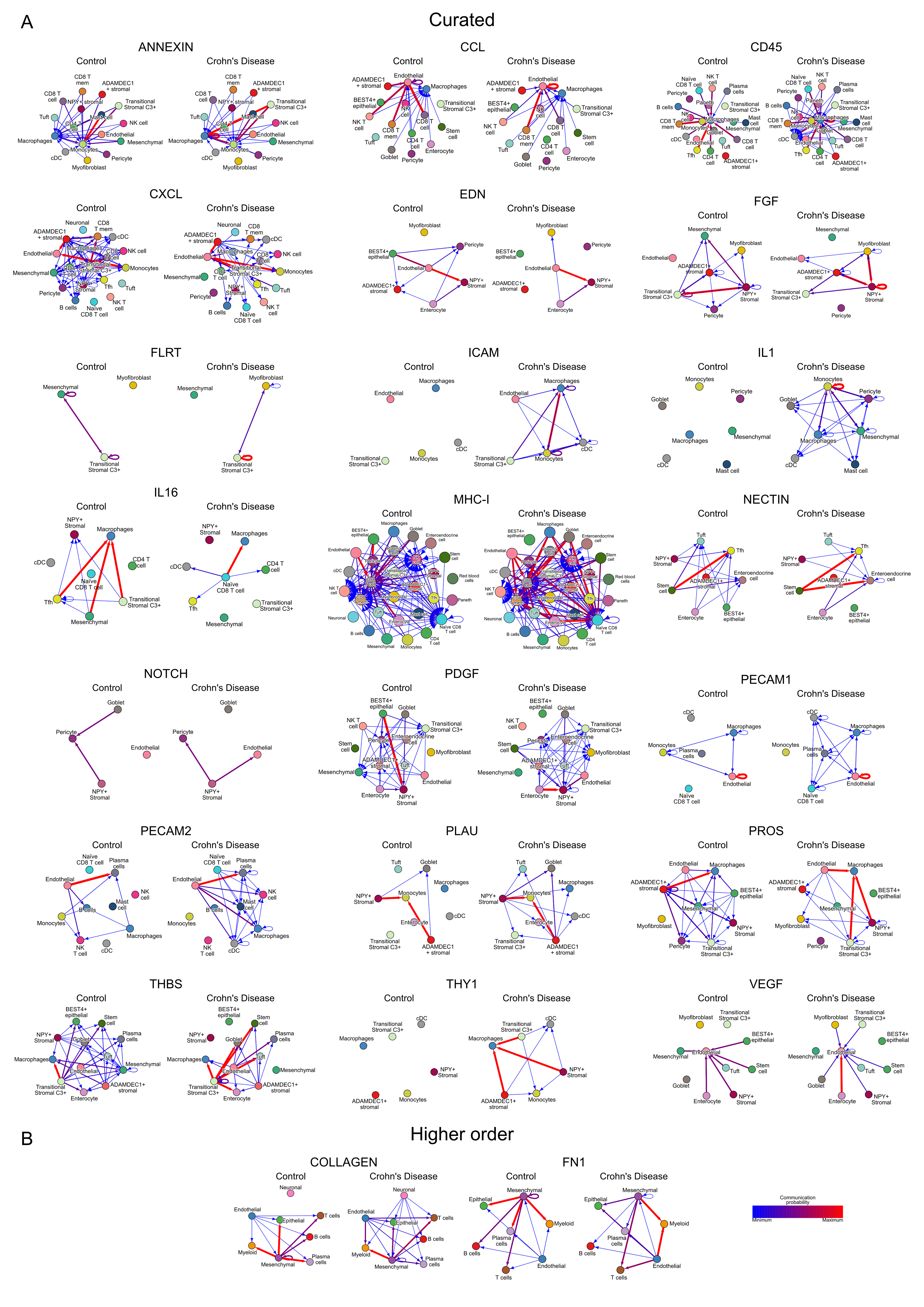


## Figure S20. Pathway communication networks between curated cell types or cell-type categories in normal control and CD FSL sample data. Network analysis of signalling pathways between individual curated cell types (A) and cell-type categories (B), constructed from directed graphs showing cell–cell communication pathways between normal control sample data (left) and CD FSL sample data (right) for each signalling pathway (subheading). The arrow direction indicates the communication pathway’s direction, and the arrow colour indicates the strength of the communication probability (blue indicating low/minimum and red indicating high/maximum).

Table S1. Archival samples details.

| Sample ID | Location | Diagnosis |
| --- | --- | --- |
| SR149206 | Ileum | Crohn's disease |
| SR149207 | Ileum | Crohn's disease |
| SR149208 | Ileum | Crohn's disease |
| SR149209 | Ileum | Crohn's disease |
| SR149210 | Ileum | Crohn's disease |
| SR149211 | Ileum | Crohn's disease |
| SR149212 | Ileum | Crohn's disease |
| SR149213 | Ileum | Crohn's disease |
| SR149214 | Ileum | Crohn's disease |
| SR149215 | Ileum | Crohn's disease |
| SR149216 | Ileum | Normal control |
| SR149217 | Ileum | Normal control |
| SR149218 | Ileum | Normal control |
| SR149219 | Ileum | Normal control |
| SR149220 | Ileum | Normal control |
| SR149221 | Ileum | Normal control |
| SR149222 | Ileum | Normal control |
| SR149223 | Ileum | Normal control |
| SR149224 | Ileum | Normal control |
| SR149225 | Ileum | Normal control |
| SR149226 | Ileum | Normal control |
| SR149227 | Ileum | Normal control |
| SR149228 | Ileum | Normal control |
| SR149229 | Ileum | Normal control |
| SR149230 | Ileum | Normal control |
| SR149231 | Ileum | Normal control |
| SR149232 | Ileum | Normal control |
| SR149233 | Ileum | Normal control |
| SR149234 | Ileum | Normal control |
| SR149235 | Ileum | Normal control |
| SR149236 | Ileum | Normal control |
| SR149237 | Ileum | Normal control |
| SR149238 | Ileum | Normal control |
| SR149239 | Ileum | Normal control |
| SR149240 | Ileum | Normal control |
| SR149241 | Ileum | Normal control |
| SR149242 | Ileum | Normal control |
| SR149243 | Ileum | Normal control |
| SR149244 | Ileum | Normal control |
| SR149245 | Ileum | Normal control |
| SR149246 | Ileum | Crohn's disease |
| SR149247 | Ileum | Crohn's disease |
| SR149248 | Ileum | Crohn's disease |
| SR149249 | Ileum | Crohn's disease |
| SR149250 | Ileum | Crohn's disease |
| SR149251 | Ileum | Crohn's disease |
| SR149252 | Ileum | Crohn's disease |
| SR149253 | Ileum | Crohn's disease |
| SR149254 | Ileum | Crohn's disease |
| SR149255 | Ileum | Crohn's disease |
| SR149256 | Ileum | Crohn's disease |
| SR149257 | Ileum | Crohn's disease |
| SR149259 | Ileum | Crohn's disease |
| SR149260 | Ileum | Crohn's disease |
| SR149262 | Ileum | Crohn's disease |
| SR149263 | Ileum | Crohn's disease |
| SR149264 | Ileum | Crohn's disease |
| SR149265 | Ileum | Crohn's disease |
| SR149266 | Ileum | Crohn's disease |
| SR149267 | Ileum | Crohn's disease |

Table S2. Immunohistochemistry conditions for specific antibodies.

| **Staining type** | **Antigen retrieval time** | **1^o^ Ab source (clone)** | **1^o^ Ab conc. (dilution factor)** | **2^o^ Ab type and source** | **2^o^ Ab conc. (dilution factor)** | **DAB time (s)** |
| --- | --- | --- | --- | --- | --- | --- |
| CD3 (General T cells) | 10 min | Rabbit anti-human (A045229-2, Agilent) | 4 mg/l (1:100) | Goat anti-rabbit (P044801-2, Agilent) | 6.67 mg/l (1:150) | 30 |
| CD4 (Helper T cells) | 15 min | Mouse anti-human (M731029-2, Agilent) | 3.21 mg/l (1:150) | Goat anti-mouse (P044701-2, Agilent) | 6.67 mg/l (1:150 | 120 |
| CD8 (Cytotoxic T cells) | 10 min | Mouse anti-human (M710301-2, Agilent) | 3.14 mg/l (1:100) | Goat anti-mouse (P044701-2, Agilent) | 6.67 mg/l (1:150) | 30 |
| Smooth Muscle Actin | 10 min | Mouse anti-human (M085129-2, Agilent) | 0.71 mg/l (1:200) | Goat anti-mouse (P044701-2, Agilent) | 6.67 mg/l (1:150) | 10 |
| CD20 (B cells) | 10 min | Mouse anti-human (M075501-2, Agilent) | 0.63 mg/l (1:200) | Goat anti-mouse (P044701-2, Agilent) | 6.67 mg/l (1:150) | 30 |
| CD31 (Endothelial cells) | 10 min | Mouse anti-human (M082301-2, Agilent) | 2.01 mg/l (1:100) | Goat anti-mouse (P044701-2, Agilent) | 6.67 mg/l (1:150) | 60 |
| CD68 (Macrophage cells) | 10 min | Mouse anti-human (M087601-2, Agilent) | 0.63 mg/l (1:200) | Goat anti-mouse (P044701-2, Agilent) | 6.67 mg/l (1:150) | 30 |

All antibodies were ordered from Agilent, Cheadle, UK.

Table S3. Mucosa correction pixel classifier settings.

| Classifier | Random trees (Rtrees) |  |
| --- | --- | --- |
| Resolution | Low (3.63 µm/px) |  |
| Features | Default multiscale features | Channels: Haematoxylin, PSR scales: 1.0 Features: Gaussian, weighted deviation |

Table S4. Collagen detection pixel classifier settings.

| Classifier | Random trees (Rtrees) |  |
| --- | --- | --- |
| Resolution | High (0.91 µm/px) |  |
| Features | Default multiscale features | Channels: Haematoxylin, PSR, residual scales: 1.0 Features: Gaussian, Weighted deviation |

Table S5. Positive cell detection settings for each antibody.

| **Stain** | **Detection image** | **Request pixel size (µm)** | **Background radius (µm)** | **Sigma (µm)** | **Minimum area (µm^2^)** | **Maximum area (µm^2^)** | **Threshold 1+** |
| --- | --- | --- | --- | --- | --- | --- | --- |
| CD3 (General T-cells) | Optical density sum | 0.7 | 10 | 1 | 10 | 400 | 0.1574 |
| CD4 (Helper T-cells) | Optical density sum | 0.7 | 10 | 1 | 10 | 400 | 0.1574 |
| CD8 (Cytotoxic T-cells) | Optical density sum | 0.7 | 10 | 1 | 10 | 400 | 0.1574 |
| Smooth Muscle Actin | Optical density sum | 0.7 | 10 | 0.5 | 10 | 500 | 0.1 |
| CD20 (B-cells) | Optical density sum | 0.7 | 10 | 1 | 10 | 400 | 0.1574 |
| CD31 (Endothelial cells) | Optical density sum | 0.7 | 10 | 1 | 10 | 400 | 0.1574 |
| CD68 (Macrophage cells) | Optical density sum | 0.7 | 10 | 1 | 10 | 400 | 0.1574 |

Table S6. Density Map function settings for CD68+ cell granulomas and CD3+ and CD20+ lymphoid aggregates.

| Object type | All detections |
| --- | --- |
| Main class | Any |
| Secondary class | Positive (inc. 1+, 2+, 3+) |
| Density type | By area (raw counts) |
| Density radius | 150 |

Table S7. Density Map threshold settings.

| Antibody Staining | Setting | Setting value |
| --- | --- | --- |
| CD68+ (macrophage granulomas) | Density threshold | 400 |
| CD3+ (T-lymphoid aggregates) | Density threshold | 300 |
| CD20+ (B-lymphoid aggregates) | Density threshold | 400 |
